# Genotoxic stressors mimicking synovial microenvironment modulate B cell fate in RA-FLS co-culture

**DOI:** 10.3389/fimmu.2026.1892572

**Published:** 2026-07-13

**Authors:** Denada Bruci, Torsten Lowin, Gerhard Fritz, Francesca Wolf, Georg Pongratz

**Affiliations:** 1 Department of Rheumatology and Hiller Research Center, University Hospital Düsseldorf, Heinrich-Heine University Düsseldorf, Düsseldorf, Germany; 2 Institute of Toxicology, Medical Faculty and University Hospital Düsseldorf, Heinrich-Heine University Düsseldorf, Düsseldorf, Germany; 3 Department of Rheumatology, Barmherzige Brüder Hospital, Regensburg, Germany

**Keywords:** B cells, DNA damage, genotoxic stress, RA-FLS, rheumatoid arthritis

## Abstract

Rheumatoid arthritis (RA) is characterized by persistent synovial inflammation in which fibroblast-like synoviocytes (RA-FLS) and B cells interact within a potentially genotoxic microenvironment. This study investigates how naive B cells respond to cytotoxic stress when supported by RA-FLS in a co-culture model, using viability assays, time resolved γ-H2AX MFI, RT-qPCR of DNA damage response and differentiation genes, and flow cytometric analysis of subsets and surface markers. Naive B cells exhibited greater loss of viability than RA-FLS, and time-resolved γ-H2AX MFI showed an earlier signal peak and decline in naive B cells versus prolonged retention in RA-FLS (0-24h post-treatment); these reflect overall damage-response dynamics rather than direct DSB repair. Importantly, γ-H2AX levels indicate DNA damage but do not directly quantify DNA break repair. Transcriptional profiling showed selective induction of ATM, APEX1, RAD50, BAX, and BCL6 following treatment, consistent with engagement of a focused DNA damage response program. Prolonged co-culture was associated with the expansion of B cell enriched compartments derived from naïve B cells, and with increased expression of stromal markers CD90 and podoplanin on both B cells and RA-FLS, while IL-10 secretion was reduced under cytotoxic conditions. Together, these findings define differential stress responses and phenotypic adaptations of B cells and RA-FLS within an inflammatory co-culture system and suggest that genotoxic stress may contribute to remodeling of immune-stromal interactions in the rheumatoid synovium.

## 1 Introduction

Rheumatoid arthritis (RA) is a chronic autoimmune disease in which immune and stromal compartments cooperate to sustain joint inflammation and tissue damage (1). Among adaptive immune cells, B lymphocytes play an essential role in the pathogenesis of RA. They produce autoantibodies, present antigens to T cells, form ectopic lymphoid structures, and modulate inflammation through cytokine production, including IL-6 and TNF-α (2). Activated B cells display a unique approach to maintaining genome integrity. These cells generate DNA lesions during V(D)J recombination, and after activation, through activation-induced cytidine deaminase (AID) during somatic hypermutation and class-switch recombination. These processes require an intact DNA damage response (DDR) to preserve genomic stability while enabling antibody diversification (3, 4). When inflammatory cues accompany these programmed breaks, checkpoint signaling can intensify, repair pathway choice may shift, and cell fate can change toward proliferation, apoptosis, or effector differentiation (3, 5, 6). Thus, DDR signaling is not only a housekeeping mechanism; it can influence B cell immune function. This intrinsic sensitivity likely contributes to the responsiveness of B cells to the conditions of their tissue niche (5, 7). The synovial niche provides such an environment. Fibroblast-like synoviocytes (FLS) line the synovial membrane and play an active role in local inflammation by delivering survival factors, co-stimulatory signals, and a distinct metabolic and redox environment (8, 9). Active RA synovium exhibits elevated levels of reactive oxygen species (ROS) and oxidative by-products from infiltrating leukocytes, FLS metabolism, hypoxia reoxygenation cycles, and iron-driven reactions (10, 11). These oxidative and genotoxic inputs are often framed as tissue injury, but for B cells, they may also act as modulators of signaling thresholds and genome maintenance (12, 13). In other words, the stromal layer may influence the same DDR pathways that govern B cell fate, particularly under conditions of chronic oxidative and genotoxic stress within the rheumatoid synovium.

To investigate how genotoxic stressors that model selected aspects of the rheumatoid arthritis synovial microenvironment modulate B cell fate within a stromal niche, we established a co-culture system of healthy human naive B cells with RA-derived FLS. We exposed the co-cultures to a single, defined dose/concentration of oxidative/genotoxic stimuli such as γ-irradiation (γ-IR), hydrogen peroxide (H_2_O_2_), and 4-hydroperoxyifosfamide (4-OOH IFA). γ-IR, encountered in radiosynoviorthesis and diagnostic imaging, among other types of DNA damage, generates clustered double-strand breaks predominantly repaired by Ku-dependent non-homologous end joining (NHEJ), alongside contributions from homologous recombination (HR) (14, 15). H_2_O_2_, a short-lived diffusible ROS generated by activated myeloid cells and inflamed stromal elements in inflamed joints, produces base modifications and DNA strand breaks that predominantly engage base-excision repair (BER), with additional involvement of NHEJ and HR (1, 16). 4-OOH IFA, the active metabolite of the alkylating agent ifosfamide and structural analogue of cyclophosphamide used in severe organ-threatening autoimmune conditions, results in DNA interstrand cross-links that require multi-step processing involving homologous recombination and associated cross-link repair pathways (17).

We assessed B cell viability, DNA damage as measured by the Ser139-phosphorylated form of the histone variant H2AX (γ-H2AX), cell cycle status using Ki-67/PI flow cytometry, and the expression of selected genes related to DNA damage, repair, and B cell differentiation (18). Following exposure, cultures were stimulated with CpG oligodeoxynucleotide (ODN2006) and anti-IgM to evaluate activation markers and functional outputs. These analyses were designed to determine how B cell survival, activation, and phenotypic adaptation are affected by different genotoxic stressors in the presence of RA-FLS as stromal support, providing descriptive data on B cell responses within a reductionist synovial-like microenvironment. In this framework, RA-FLS primarily define the tissue context rather than constituting the main object of analysis; DNA damage related transcriptional responses and functional readouts in RA-FLS monocultures and RA-FLS-PBMC co-cultures exposed to comparable sublethal genotoxic stress have been characterized in our previous study and are therefore not duplicated here (19). Accordingly, our experimental panel in the present study is focused on how distinct, clinically relevant DNA damage modalities impact B cell survival, activation, and fate within an RA-FLS-dependent synovial-like niche. A deeper understanding of B-cell adaptation to genotoxic stress in RA may help to generate hypotheses about treatment strategies and immune recovery following joint inflammation or clinical exposures such as imaging and immunosuppressive therapy (20).

## 2 Materials and methods

### 2.1 Establishment of cell culture and co-culture systems

Fibroblast-like synoviocytes (FLS) were derived from synovial specimens of rheumatoid arthritis (RA) patients following informed consent and institutional ethical approval (Study-Nr.: 2022-2189_7) (21). Mycoplasma negativity was confirmed routinely for all cell cultures. RA-FLS were expanded to passages 3 to 5 and plated at a density of 1 × 10^5^ cells per well in 48-well plates. Culture conditions employed RPMI 1640 medium (Sigma-Aldrich) supplemented with 10% fetal bovine serum (FBS; Gibco), 1% GlutaMAX (Thermo Fisher Scientific), 1% sodium pyruvate (Thermo Fisher Scientific), 1% penicillin-streptomycin (Thermo Fisher Scientific), and 10 mM HEPES (Thermo Fisher Scientific), maintained at 37 °C in a humidified incubator with 5% CO_2_. Buffy coats from healthy adult donors were obtained from the blood bank of the Heinrich Heine University Hospital Düsseldorf. After 24 hours of RA-FLS initial adherence, peripheral blood mononuclear cells (PBMCs) were isolated from buffy coat donors using Lymphoprep density-gradient centrifugation (Progen), washed, and counted. B cells were then isolated from PBMCs using the Naive B cell Isolation Kit (Miltenyi Biotec) for all experiments except the Annexin V vs PI assay, for which a Pan B Cell Isolation Kit (Miltenyi Biotec) was used. Purified B cells/naive B cells were added to RA-FLS cultures at a ratio of 5:1 (B cells: RA-FLS) in identical medium. Purity of naive B cells isolation was checked before every experiment via flow cytometry (see **Suplementary Figure 9A**).

### 2.2 Genotoxic treatments and stimulation

Experimental groups included (1) B cells/naive B cells alone, which were subjected to γ-irradiation (0–4 Gy; Gammacell 1000 Elite, Nordion International) prior to culture and then added to untreated RA-FLS, and (2) co-cultures of B cells/naive B cells with RA-FLS, which were treated with 4-OOH IFA (0-34 µM; Niomech IIT GmbH) or hydrogen peroxide (0-160 µM; Sigma-Aldrich). Half of the wells were collected 24 h after treatment for various assays. After 24 hours, CpG ODN 2006 (5 µg/mL; Invivogen) and α-IgM (10 µg/mL; Jackson ImmunoResearch) were added directly to the co-culture, and co-cultures continued until day 9 (8 days post treatment). The experimental workflow is detailed in **Supplementary Figure 1**. All reagents and antibodies used are listed in **Supplementary Table 1**. Treatment conditions were randomized across wells of multi-well plates to avoid positional batch effects; no row or column was dedicated to a single condition.

### 2.3 Assessment of cell viability

Viability was measured using an Annexin V-FITC/propidium iodide (PI) flow cytometry assay (Miltenyi Biotec) according to the manufacturer’s instructions, with data acquisition on a CytoFLEX LX flow cytometer (Beckman Coulter) after 24 h of treatment (see **Supplementary Figure 2** for gating strategy). Annexin V^-^/PI^-^ cells were defined as viable cells, and these values were used to generate dose/concentration-response curves for γ−IR (0–4 Gy), 4−OOH IFA (0-34 µM), and H_2_O_2_ (0-160 µM). Relative IC_50_ values were calculated using four−parameter logistic regression (**Supplementary Table 2**). Relative IC_20_/IC_50_ concentrations determined at 24 h for naive B cells were approximately 0.58 Gy/1.3 Gy for γ−IR, 3.5 µM/7.8 µM for 4−OOH IFA, and 24 µM/35 µM for H_2_O_2_. Unless specified otherwise, IC_20_ and IC_50_ throughout denote operational relative effect levels, defined as the concentrations producing 20% and 50% of the maximal viability loss observed across the dose/concentration-response range for naive B cells, relative to vehicle−treated controls, and are used as sublethal benchmarks rather than absolute cytotoxicity thresholds. A comprehensive list of reagents and antibodies appears in **Supplementary Table 1**.

### 2.4 Real-time quantitative PCR

For mRNA quantification, total RNA was isolated from either co-cultured cells, naive B cells-RA-FLS, or RA-FLS monocultures at 24 hours and 5 days post-exposure, using the NucleoSpin RNA isolation kit (Macherey-Nagel), adhering to the manufacturer’s protocol. Reverse transcription was conducted using the High-Capacity cDNA Reverse Transcription Kit (Applied Biosystems). Target gene expression, including BCL6, ATM, AICDA, and others, was determined by qPCR using SYBR Green Master Mix (Applied Biosystems), with 18S rRNA as the internal reference. Relative gene quantities were calculated using the ΔΔCt method. Primer sequences were identical to those used in our previous study and are listed in **Supplementary Table 3** for completeness (19). RA-FLS 24-hour monoculture qPCR data were generated and reported in our previous study (Bruci et al., 2026) and are not re-shown here; any immunoglobulin or AICDA/germinal center factor measurements in the present work therefore refer to co-cultures and serve to document baseline stromal transcriptional responses in that context (19).

### 2.5 Flow cytometric assays

*General Sample Processing*: Cells were enzymatically detached using Accutase+ EDTA for 10 minutes at 37 °C, rinsed in FACS buffer (PBS containing 3% FBS and 2 mM EDTA), then subjected to FcR receptor blockade (Miltenyi, dilution 1:5) for 15 minutes at 4 °C. Surface antigen staining was conducted for 20 minutes at room temperature with the following antibodies: CD19 eFluor 405 (Invitrogen, 1:20), CD27-VioBright R720 (1:50) and CD90-PerCP-Vio700 (Miltenyi, 1:50). Following staining, cells were fixed in 4% paraformaldehyde (Thermo Fisher Scientific; 15 minutes, RT), permeabilized in 0.1% Triton X-100 (Thermo Fisher Scientific; 10 minutes, RT), and subsequently stained for intracellular markers in PBS supplemented with 0.05% Tween-20 and 3% FBS. Data acquisition utilized a CytoFLEX LX cytometer (Beckman Coulter), and analysis was performed using FlowJo v10.8.1. All reagents and antibodies are listed in **Supplementary Table 1**. Naive B cells were defined as CD19^+^CD27^-^ lymphocytes, and memory B cells as CD19^+^CD27^+^, whereas RA−FLS were identified as CD90^+^ stromal cells. Because prolonged co−culture induced acquisition of CD90 and podoplanin on CD19^+^CD27^-^ B cell gates at late time points, naive B cells in functional flow assays (Ki−67/PI assay, phenotypic characterization assay) refer to CD19^+^CD27^-^ B cell-derived populations, which can co−express stromal markers under these conditions, whereas in early readouts (γ-H2AX and viability assays) they correspond to classical CD19^+^CD27^-^ and CD19^+^CD27^+^ B cell populations that have not yet acquired stromal markers.

#### 2.5.1 γ-H2AX DNA damage assessment

Upon completion of surface staining, fixation, and permeabilization, cells were incubated with anti-γ-H2AX-Alexa Fluor 488 antibody (BD Biosciences, 1:20) at room temperature for one hour. After washing, samples were resuspended in FACS buffer. The gating strategy for analysis is outlined in **Supplementary Figure 3**.

#### 2.5.2 Ki-67 and PI cell cycle assay

Permeabilized cells were stained with Ki-67-FITC (Miltenyi Biotec, 1:50) for 20 minutes in the dark, followed by counterstaining with propidium iodide (PI, Miltenyi Biotec, 1:100) for an additional 20 minutes at room temperature in the dark. The gating approach is described in **Supplementary Figure 4**. Comprehensive lists of all reagents and antibodies are provided in **Supplementary Table 1**.

### 2.6 Quantification of cytokines and immunoglobulins

To evaluate the functional competence of viable cells post-genotoxic challenge, supernatants were collected on day 9 (8 days post-treatment) from co-cultures exposed to γ-IR, 4-OOH IFA, or H_2_O_2_. Cytokine and chemokine concentrations (e.g., IL-10, IFN-γ, APRIL, TACI, IL-4) were analyzed using ELISA kits from R&D Systems and BD Biosciences, while immunoglobulin isotypes (IgM, IgG, IgA, IgE) were quantified using kits from StemCell Technologies. Assays were performed according to the manufacturer’s guidelines, with absorbance readings at 450 nm on a Tecan Infinite M200 Pro microplate reader (Tecan), and data were processed using Microplate Manager software (Bio-Rad). All ELISA and immunoglobulin assays included internal standards and positive control samples, which yielded signals within the expected dynamic range and confirmed assay sensitivity for the reported analytes. Results are reported as percentages relative to CpG + a-IgM-stimulated, non-genotoxic control cultures and are mapped to the corresponding viability metrics from the same wells at day 9. The complete list of kits is available in **Supplementary Table 1**.

### 2.7 Flow cytometric analysis of B cell subsets following CpG and α-IgM stimulation

After 8 days post-treatment, 7 days post-stimulation with CpG ODN2006 and α-IgM, B cell subsets in co-culture were analyzed by flow cytometry. Cells were processed as described above, omitting fixation to preserve surface antigen integrity. Following FcR receptor blockade (Miltenyi, 1:5, 15 minutes, 4 °C), surface staining was performed for 20 minutes at room temperature using the following antibodies: CD19-eFluor 450, CD27-APC, CD90-PerCP/Vio700, Podoplanin-PerCP, CD138-PE/Vio770, CD1d-BV786, CD5-PE, IgM-Alexa Fluor 700, IgD-PE/CF594, IgG-APC/Vio770, CD11c-BV605 and CD38-PE/Cy5. Zombie UV Fixable Viability Dye was included to exclude dead cells (>85% viability post-dissociation). Controls comprised isotype-matched antibodies (matched species/isotype/fluorophore/concentration), unstained samples, single-color-stained cells (for compensation matrix calculation), and RA-FLS monocultures (confirming CD19, CD27, CD38, CD138, IgM, IgG, IgD negativity). Antibody clones, fluorochromes, and suppliers are detailed in **Supplementary Table 1**. Data acquisition was performed on a CytoFLEX LX cytometer (Beckman Coulter) and analyzed using FlowJo v10.8.1. Gating strategies were established using isotype/unstained/RA-FLS controls; representative gating, including monoculture overlays, is shown in **Supplementary Figures 5** and **9**. At these late time points, the populations gated as naive B cells, class−switched memory B cells and short−lived plasma-like cells are therefore used as operational labels for B cell−derived CD90^+^podoplanin^+^ aggregates identified according to the B cell gating strategy shown in **Supplementary Figure 5**, rather than as lineage−pure subsets.

### 2.8 Cell dissociation methods

For dissociation optimization on day 9, co-cultures were washed with pre-warmed PBS and subjected to multiple enzymatic and chelation-based conditions. The wells were incubated with 1X trypsin-EDTA (ThermoFisher) for 10 min at 37 °C, after which the reaction was stopped with complete medium. Additional wells were incubated for 25 min at 37 °C with Liberase (0.8 mg/mL, Sigma-Aldrich) alone, Dispase II (3 mg/mL, Thermofisher Scientific) together with citrate buffer (Thermo Fisher), citrate buffer alone, citrate buffer plus EDTA (Invitrogen, 4 mM), cold PBS (Sigma), Accutase (Sigma-Aldrich), Accutase plus EDTA (4 mM), or a cocktail of DNase I (0.05 μg/mL, Stemcell), Liberase (0.8 mg/mL) and Dispase II (3 mg/mL) with or without subsequent EDTA treatment. For citrate buffer-based detachment, cells were washed once in PBS, incubated for 10-15 min at 37 °C in pre-warmed working citrate buffer prepared by diluting a 10X stock solution [1.35 M potassium chloride (Merck) + 0.15 M tri-sodium citrate dihydrate (Carl Roth)] 1:10 in ddH_2_O immediately before use, and then mechanically triturated. In all conditions involving sequential chelation, EDTA was applied as a separate 10-min incubation at 37 °C immediately after removal of the initial solution (citrate buffer, PBS, Accutase, or the DNase I/Liberase/Dispase II cocktail). All reactions were quenched with complete medium, cells were gently pipetted, washed in staining buffer, and analyzed by flow cytometry as shown in **Supplementary Figure 9C**. For selected co−culture experiments, naive B cells were pre−labeled with CellTrace CFSE (Thermo Fisher Scientific, 1:1000) for 10 min at 37 °C in serum−free medium, followed by quenching with complete medium and extensive washing, to enable tracking of B cells after genotoxic treatment in subsequent co−culture.

### 2.9 Statistical analysis

Data are presented as mean ± standard error of the mean (SEM). Repeated measures one-way ANOVA with Dunnett’s multiple comparisons test was applied for viability, DNA damage, cell cycle distribution, cytokine/chemokine/immunoglobulin secretion (day 9), and RT-qPCR datasets to accommodate the paired design across conditions from matched donor samples. Normality was verified by the Shapiro-Wilk test (p > 0.05), and homogeneity of variance was confirmed via the Brown-Forsythe test (p > 0.05) and visual inspection of SEM overlays. To account for multiple testing across conditions, cell pairings, and longitudinal time points, p-values from ANOVA were subjected to Dunnett’s post-hoc correction for multiple comparisons versus the corresponding control condition, while the Bonferroni adjustment was applied for targeted head-to-head pairwise comparisons between specific cell types and individual treatment groups at discrete time points. Within-donor variability is depicted as SEM, with between-donor variation controlled through a repeated measures design. No additional global adjustment across different endpoints (e.g., across all genes and cytokines jointly) was applied, and results are therefore interpreted with an emphasis on concordant, biologically coherent patterns across donors and related readouts rather than on isolated marginally significant comparisons. Statistical significance denoted as: *p ≤ 0.05, **p ≤ 0.01, ***p ≤ 0.001, ****p ≤ 0.0001, ns, not significant. In all analyses, N denotes the number of independent biological replicates (donors), and n denotes the number of technical replicates per condition; the specific N and n for each experiment are reported in the corresponding figure legends. All analyses were conducted in GraphPad Prism 8 using independent biological replicates (N = 3–6 donors per experiment).

### 2.10 Language editing

The language and grammar of this manuscript were checked using language editing assistance, while all content, data interpretation, and conclusions were generated solely by the authors.

## 3 Results

### 3.1 Genotoxic stress induces distinct viability responses in naive and memory B cells

**Figure 1:**
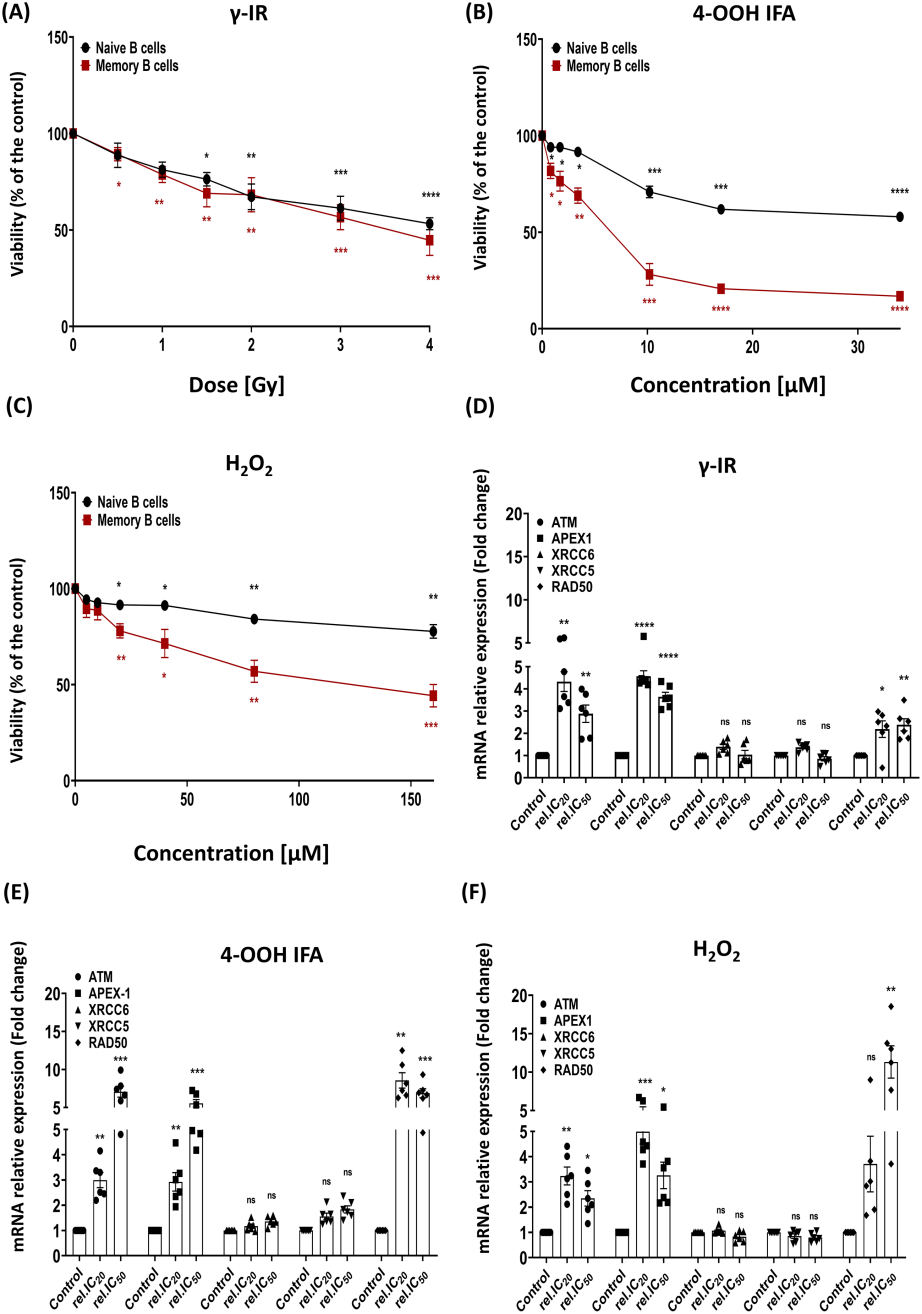
Single dose/concentration genotoxic stress differentially affects viability and early DNA damage responses of B cell subsets in co-culture. Dose/concentration response of viable (Annexin V^-^/PI^-^) naive B cells (black), memory B cells (red) within B cell-RA-FLS co-cultures 24 h after a single exposure to **(A)** γ-irradiation (0–4 Gy), **(B)** 4-OOH IFA (0-34 µM), or **(C)** H_2_O_2_ (0-160 µM). Panels **(D–F)** show relative mRNA expression of DNA damage response and repair genes ATM, APEX1, XRCC5, XRCC6 and RAD50 in naive B cell-RA-FLS co-cultures 24 h after treatment with γ-IR **(D)**, 4-OOH IFA **(E)**, or H_2_O_2_ **(F)** at relative IC_20_ and relative IC_50_ dose/concentrations, displayed as fold change over untreated controls. Four-parameter logistic curves were fitted to determine relative IC_20_ and IC_50_ dose/concentrations for each condition (see **Supplementary Table 2** for estimated values). In all panels, rel. IC_20_ and rel.IC_50_ denote relative effect levels that are used as sublethal benchmarks, as defined in the Methods. All data are presented as mean ± SEM with one value per donor and dose/concentration, expressed as percentage or fold change relative to the corresponding untreated control. Group differences versus untreated control were analyzed using repeated-measures one-way ANOVA with Dunnett’s post-hoc test; *p ≤ 0.05, **p ≤ 0.01, ***p ≤ 0.001, ****p ≤ 0.0001, ns: not significant. B cell-RA-FLS co-cultures (viability, A-C): N = 3 donors, n = 2; naive B cell-RA-FLS co-cultures (RT-qPCR, D-F): N = 3 donors, n = 2.

To characterize the exposure response, we quantified viability using an Annexin V/PI assay (Annexin V-/PI- as viable cells) 24 hours after single graded treatments. The viability was quantified within naive and memory B cell subsets (CD19^+^CD27^-^/CD19^+^CD27^+^) in the B cell-RA−FLS co−culture via flow cytometry. Treatments followed a differential exposure protocol: B cells received γ-IR (Gy) before co-culture initiation, then added to untreated RA-FLS monolayers, whereas 4-OOH IFA or H_2_O_2_ were administered directly to established co-cultures. γ-IR induced a monotonic decline in viability in both naive and memory B cells within co-culture (**Figure 1A**). The monotonic decrease in viability following γ-IR was significant at all tested concentrations in memory B cells up to 4 Gy. In contrast, in naive B cells, loss of viability reached statistical significance at 1.5 Gy, indicating differing sensitivity profiles between subsets (**Figure 1A**; **Supplementary Table 2**). 4-OOH IFA exerted the most pronounced cytotoxic effect, particularly in memory B cells (relative IC_50_, 5 µM), with significant reductions evident at concentrations ≥10 µM (**Figure 1B**; **Supplementary Table 2**). Naive B cells were moderately more resistant (relative IC_50_, 7.8 µM), whereas, as reported previously by Bruci et al. (2026), RA-FLS monocultures maintained more than 50% viability across the same 4-OOH IFA concentration range, with an IC_50_ of approximately 7.7 µM, which may suggest that the doses used here are more toxic for B cells than for stromal cells (19). For hydrogen peroxide, viability loss was concentration-dependent across B cell subsets. Statistically significant reductions were observed from 20 µM in memory B cells (relative IC_50_, 35 µM), while naive B cells exhibited greater resistance (relative IC_50_, 78 µM) (**Figure 1C**; **Supplementary Table 2**). By contrast, RA-FLS monocultures showed minimal susceptibility in our prior study, with viability remaining above 80% even at the highest H_2_O_2_ concentrations tested, further supporting a relative resistance of RA-FLS to oxidative stress under the sublethal conditions used here (19). IC_20_ and IC_50_ doses/concentrations, defined as the doses/concentrations reducing naive B cell viability by approximately 20% and 50% relative to vehicle−treated controls within the dose/concentration range, were therefore used as sublethal benchmark conditions to allow functional comparisons across stimuli while maintaining sufficient viable cells for downstream assays. As these doses/concentrations were sublethal for RA-FLS but clearly impacted B-cell viability, and revealed distinct sensitivity profiles between naive and memory B cells, subsequent DNA damage analyses focused on naive B cell-RA-FLS co-cultures at the predefined relative IC_20_ and IC_50_ doses/concentrations.

### 3.2 Sublethal genotoxic stress selectively modulates DNA damage sensor and apoptotic gene expression in naive B cell-RA-FLS co-cultures

To further characterize the observed differential sensitivity, we next analyzed DNA damage sensor activation and apoptotic gene regulation in bulk naive B cell-RA-FLS co-cultures under the determined relative IC_50_ and IC_20_ (**Figures 1D–F**; **Supplementary Figure 6**). At 24 hours, both γ-IR and H_2_O_2_ induced a comparable pattern of DNA damage sensor activation, with ATM and APEX1 increasing approximately 3 to 5-fold at both IC_20_ and IC_50_. 4-OOH IFA produced a stronger transcriptional response for these genes, reaching ~8 to 9-fold elevation at IC_50_. RAD50 expression was upregulated by ~2 to 3-fold after γ-IR (IC_20_ and IC_50_), ~10-fold in response to 4-OOH IFA (IC_20_ and IC_50_), and at IC_50_ after H_2_O_2_ exposure. No significant changes were observed for XRCC6, XRCC5, and BRCA1 across treatments (**Figures 1D–F**; **Supplementary Figures 6A–C**). In contrast, BRCA2 was downregulated at H_2_O_2_ IC_50_. BCL6 expression was upregulated 4 to 6−fold after γ−IR, and was also increased at 4−OOH IFA IC_50_, with no change observed under the remaining conditions. TNFSF13B expression increased 2 to 3-fold in response to γ-IR (IC_20_ and IC_50_) with no changes across other treatments. RAD51 transcripts decreased only at the H_2_O_2_ IC_50_ treatment, without changes in other conditions (**Supplementary Figures 6A–C**). TP53 transcripts decreased following H_2_O_2_ treatments at IC_20_ and IC_50_, and 4-OOH IFA, γ-IR at IC_50_. No significant modulation was observed for CDKN1A (p21), FAS, or FASLG transcripts across any treatment or concentration, with the exception of a downregulation of FASLG at H_2_O_2_ IC_50_ (**Supplementary Figures 6D–F**). BAX transcripts increased approximately 5 to 15−fold following γ−irradiation (γ−IR) and H_2_O_2_ treatments at both IC_20_ and IC_50_ concentrations, whereas 4−OOH IFA induced BAX upregulation only at IC_50_, with no significant change at IC_20_. BCL2 transcripts increased only under γ-IR IC_50_ treatment (**Supplementary Figures 6D–F**). Consistent with these patterns, the previously published RA−FLS monoculture study (Bruci et al., 2026) showed a more restricted transcriptional response to sublethal genotoxic stress, with H_2_O_2_ selectively increasing XRCC5 and inducing BCL6, BAX, and TP53 at IC_20_ but downregulating BAX and BRCA2 at IC_50_, and 4−OOH IFA elevating APEX1, ATM, RAD50, XRCC5, RAD51, and apoptotic regulators such as BAX, FAS, FASLG, PDCD1, BCL6, TP53, and BRCA2 in a concentration-dependent manner (19).

Taken together, these findings indicate that genotoxic stress transcriptional responses in the naive B cell/RA-FLS system at the bulk co-culture level might be primarily limited to selected DNA damage sensor and apoptotic genes (**Figures 1D–F**; **Supplementary Figures 6A–F**), while RA-FLS alone exhibits a narrower but stimulus-specific modulation of DNA damage and apoptotic genes under the same sublethal conditions (19). At this stage, these data reflect changes at the whole-co-culture level rather than cell-type-resolved regulation, and broader downstream effector genes were not significantly modulated. This prompted a protein-level analysis of DNA damage dynamics (γ-H2AX) in individual lineages.

### 3.3 Naive B cells and RA-FLS display distinct lineage-specific γ-H2AX profiles over time after DNA-damaging treatments

**Figure 2:**
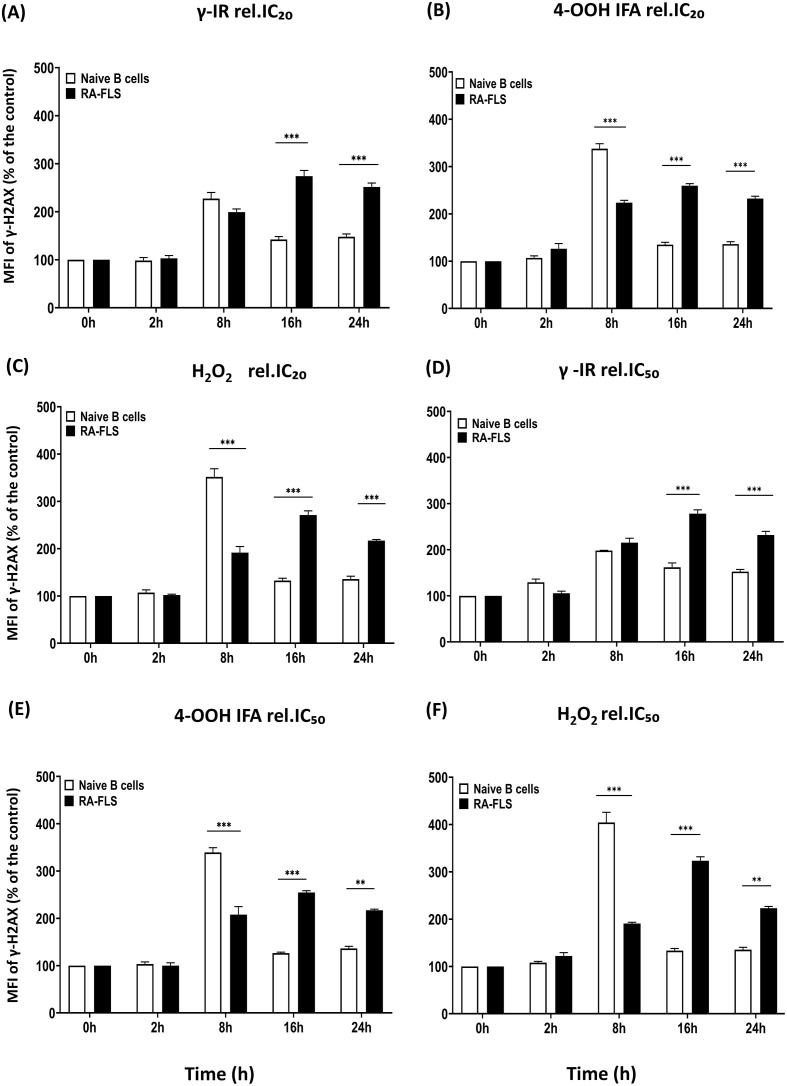
Genotoxic stress induces time-dependent γ-H2AX accumulation in naive B cell-RA-FLS co-cultures at rel.IC_20_ and rel.IC_50_. γ-H2AX median fluorescence intensity (MFI) was quantified in naive B cells and RA-FLS within naive B cell-RA-FLS co-cultures over time (0, 2, 8, 16, 24 h) following treatment with γ-IR **(A, D)**, 4-OOH IFA **(B, E)** or H_2_O_2_ **(C, F)** at rel.IC_20_ **(A–C)** or rel.IC_50_ **(D–F)** dose/concentrations derived from **Figure 1** (see **Supplementary Table 2**). In all panels, rel. IC_20_ and rel.IC_50_ denote relative effect levels that are used as sublethal benchmarks, as defined in the Methods. MFI values are expressed as a percentage of the time-matched untreated control. All data are presented as mean ± SEM with one value per donor and dose/concentration, expressed as percentage or fold change relative to the corresponding untreated control. Group differences were analyzed via repeated‑measures one-way ANOVA followed by Dunnett’s or Bonferroni’s post‑hoc tests for multiple comparisons, as appropriate; *p ≤ 0.05, **p ≤ 0.01, ***p ≤ 0.001, ****p ≤ 0.0001, ns: not significant. Naive B cell-RA-FLS co-cultures: N = 4 donors, n = 1.

After determining relative IC_20_ and IC_50_ exposure thresholds based on Annexin V vs PI assay, we next characterized DNA damage in naive B cells-RA-FLS co-cultures. γ-H2AX MFI was quantified within CD19^+^CD27^-^ naive B cell gates and CD90^+^ RA-FLS gates (**Supplementary Figure 3**). Cell types were selected to enable focused interrogation of lineage-intrinsic responses, with naive B cells chosen to minimize heterogeneity related to memory differentiation and antigen experience. Time-course analysis showed that all genotoxic exposures, γ-IR, 4-OOH IFA, and H_2_O_2_, induced an increase in γ-H2AX MFI, with higher values at 8 hours in naive B cells and 16 hours in RA-FLS (**Figure 2**; **Supplementary Figure 7**). At later time points, γ-H2AX MFI values declined in both lineages, and by 24 hours signals were lower than at their respective earlier maxima. Statistically significant increases persisted at 8h, 16, and 24 hours for both cell types and concentrations, with RA-FLS consistently displaying the highest MFI. Untreated baseline control MFI for RA-FLS was substantially greater than for naive B cells, necessitating normalization to percent of control for intra-lineage comparisons (**Supplementary Figure 8E**). Within naive B cells, a higher γ-H2AX peak was observed at 8 hours for 4-OOH IFA and H_2_O_2_ relative to γ-IR. In contrast, at 24 hours, γ-IR surpassed the other agents (**Supplementary Figures 8A, C**). By comparison, RA-FLS showed no significant differences among treatments at any time point, with the exception of a modestly higher γ−H2AX MFI for H_2_O_2_ compared with 4−OOH IFA at 16 hours, whereas neither condition differed significantly from γ−IR (**Supplementary Figures 8B, D**). Taken together, these time-resolved measurements describe distinct lineage-specific γ-H2AX MFI patterns: naïve B cells show higher γ-H2AX MFI at earlier time points, whereas RA-FLS have comparatively higher γ-H2AX MFI within the 16-24-hour window following genotoxic stress. However, γ−H2AX is an indirect marker of DNA damage and repair; without direct quantification of DNA strand breaks or repair pathway activity, we do not infer relative repair capacity or detailed kinetics from these data, and the observed γ-H2AX profiles are interpreted descriptively rather than as evidence of different repair kinetics. To complement DNA damage dynamics, we next assessed cell cycle proliferation (Ki-67) and death (propidium iodide, PI) markers at 48 and 72 hours post-treatment to evaluate longer-term cellular outcomes.

### 3.4 Naive B cell-stromal aggregates and RA-FLS undergo transient cell-cycle redistribution after genotoxic treatment

**Figure 3:**
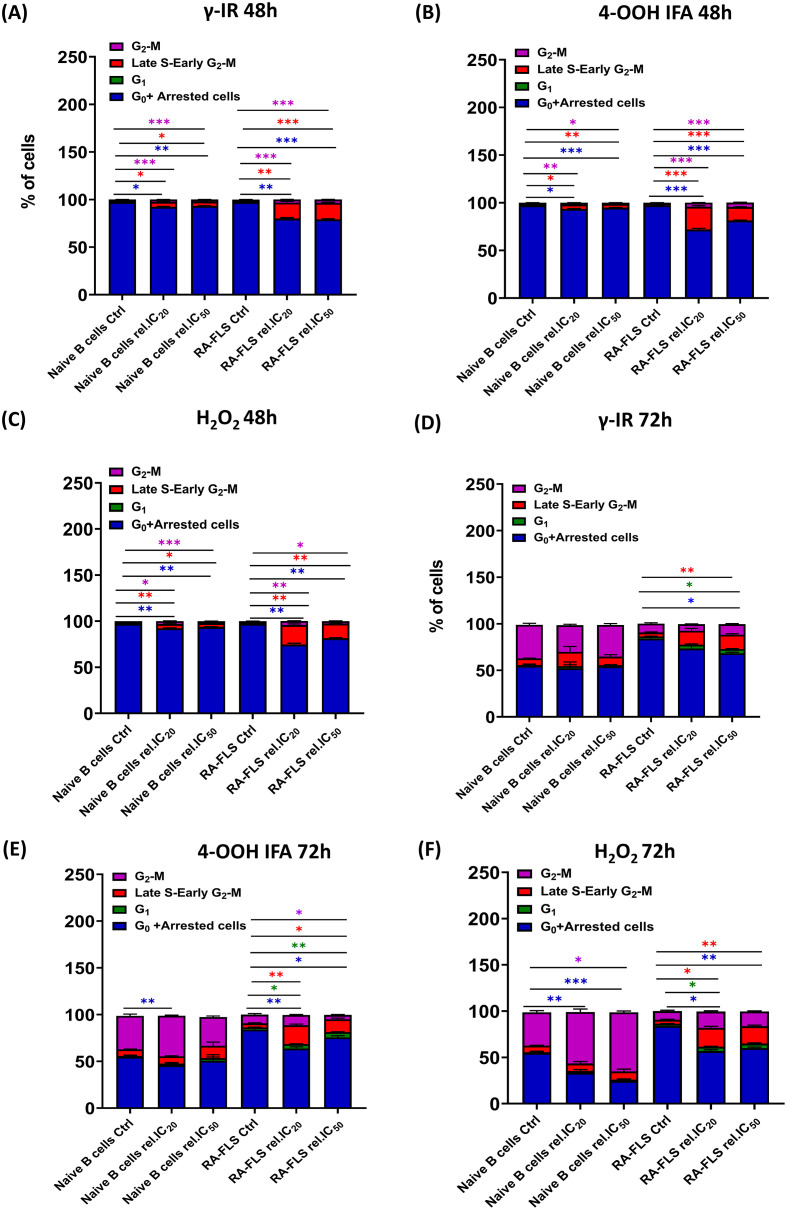
Sublethal genotoxic stress reshapes cell cycle distribution in naive B cell-RA-FLS co-cultures. Stacked bar plots show the percentages of cells in G_0_ (blue), G_1_ (green), late S-early G_2_-M (orange-red) and G_2_-M (magenta) phases in naive B cells and RA-FLS 48 h **(A–C)** and 72 h **(D–F)** after treatment with γ-IR **(A, D)**, 4-OOH IFA **(B, E)** or H_2_O_2_ **(C, F)** at rel.IC_20_ or rel.IC_50_ concentrations defined in **Figure 1** (see **Supplementary Table 2**). In all panels, rel. IC_20_ and rel.IC_50_ denote relative effect levels that are used as sublethal benchmarks, as defined in the Methods. Naive B cells and RA-FLS were gated within naive B cell-RA-FLS co-cultures and analyzed by Ki-67propidium iodide staining to resolve proliferative and quiescent compartments. In this context, naive B cells denote the CD19^+^CD27^-^CD90^+^ B cell-derived naive B cell-stromal aggregate compartment, whereas RA-FLS denotes CD19^-^CD27^-^CD90^+^ stromal cells (see **Supplementary Figure 4**). All data are presented as mean ± SEM with one value per donor and dose/concentration, expressed as percentage or fold change relative to the corresponding untreated control. Group differences versus untreated control were analyzed using repeated-measures one-way ANOVA with Dunnett’s post-hoc test; *p ≤ 0.05, **p ≤ 0.01, ***p ≤ 0.001, ****p ≤ 0.000, ns: not significant. Naive B cell-RA-FLS co-cultures: N = 4 donors, n=1.

To determine how DNA damage activation influences cell cycle progression in the naive B cell/RA-FLS co-culture model, we analyzed Ki-67-FITC/PI staining at 48 h and 72 h post-treatment (24 h and 48 h following CpG + α-IgM stimulation). Ki-67 expression was measured within CD19^+^CD27^-^ (naive B cell) and CD19^-^CD27^-^ CD90+ (RA-FLS) gates, as defined in the flow cytometry methods section. By 48 h post-treatment, CD19^+^CD27^-^ events had already acquired uniform CD90 expression in co-culture, indicating tight association with, or acquisition of stromal features from, RA-FLS; thus, the Ki-67/DNA profiles at 48 h and 72 h should be interpreted as properties of a CD19^+^CD27^-^ B cell-derived CD90^+^ compartment rather than strictly resting naive B cell-intrinsic responses, and in the context of this assay this CD19^+^CD27^-^CD90^+^ population is referred to as naive B cell-stromal aggregates (**Supplementary Figure 4**) and, for brevity, is labeled as naive B cells in the corresponding figures as specified in the figure legends. Cell cycle phases were defined based on Ki-67 expression and DNA content (PI staining); the G_0_ + arrested cell population (Ki-67^-^) encompasses both quiescent cells (2N DNA) and cells (4N DNA content), which cannot be distinguished as truly arrested versus quiescent by Ki-67 and PI staining alone (**Supplementary Figure 4**). Although these subpopulations could theoretically be separated by PI intensity, the G_0_ + arrested compartment showed uniformly high proportions across all conditions at 48 h with no treatment-specific alterations. In this study, the term G_0_ + arrested is therefore used as an operational label based on Ki-67 negativity and DNA content, and should not be interpreted as definitive proof of irreversible cell-cycle arrest. Moreover, changes observed at 72 h occurred even in RA-FLS during γ-IR experiments, where only naive B cells were directly irradiated, suggesting co-culture dynamics rather than direct cell arrest in this compartment. Therefore, Ki-67^-^ cells were analyzed collectively, with treatment-dependent redistribution assessed within the Ki-67^+^ (cycling) fractions. At 48 h, for all treatment groups and exposure levels (IC_20_ and IC_50_), both naive B cell-stromal aggregates and RA-FLS exhibited redistribution from G_0_ + arrested into cycling phases, characterized by marked enrichment in late S-early G_2_-M and G_2_-M fractions, accompanied by decreased G_0_+ arrested proportions relative to untreated, stimulated controls (**Figures 3A–C**). By 72 h post-treatment, the proportion of G_0_ + arrested cells began to decrease across all conditions, indicating partial resolution and return to baseline cycling activity. At this later time point, RA-FLS maintained significant alterations, specifically under γ-IR IC_50_, with elevated G_1_ and late S-early G_2_-M populations (**Figure 3D**). Under 4-OOH IFA exposure, RA-FLS at both IC_20_ and IC_50_ displayed increased G_1_ and late S-early G_2_-M, whereas naive B cell-stromal aggregates showed only a modest reduction in the G_0_ + arrested fraction at IC_20_ and otherwise approximated baseline distributions. For H_2_O_2_, naive B cell-stromal aggregates exhibited a G_2_-M increase at IC_50_, while RA-FLS displayed higher late S-early G_2_-M frequencies at both IC_20_ and IC_50_ and an additional G_1_ elevation under IC_20_ exposure. Altogether, these findings indicate that both naive B cell-stromal aggregates (CD19^+^CD27^-^CD90^+^ compartment) and RA-FLS (CD19^-^CD27^-^CD90^+^ compartment) might undergo transient cell cycle redistribution following genotoxic stress, with dominant accumulation in late S-early G_2_-M phases at intermediate times (48 h) and partial resolution thereafter. The stromal compartment shows more persistent changes in cell-cycle distribution at later time points, which, in this descriptive framework, are interpreted as longer-lasting deviations from baseline rather than as evidence of a stable cell-cycle arrest.

### 3.5 Naive B cell-RA-FLS co-cultures show selective, time-dependent modulation of B cell differentiation genes

Building upon DNA damage response (DDR) gene activation and cell cycle modulation described above, we next examined lineage-specific differentiation gene expression at the co-culture level to determine whether DNA damage signaling coincided with changes in B cell-linked programs. Focusing on whole naive B cell/RA-FLS co-cultures, we quantified key B cell lineage and plasma cell transcription factors in bulk co-culture samples at 24 hours and 5 days post-treatment after exposure to IC_20_ and IC_50_ concentrations of γ-IR, 4-OOH IFA, and H_2_O_2_ from the Annexin V vs PI assay.

**Figure 4:**
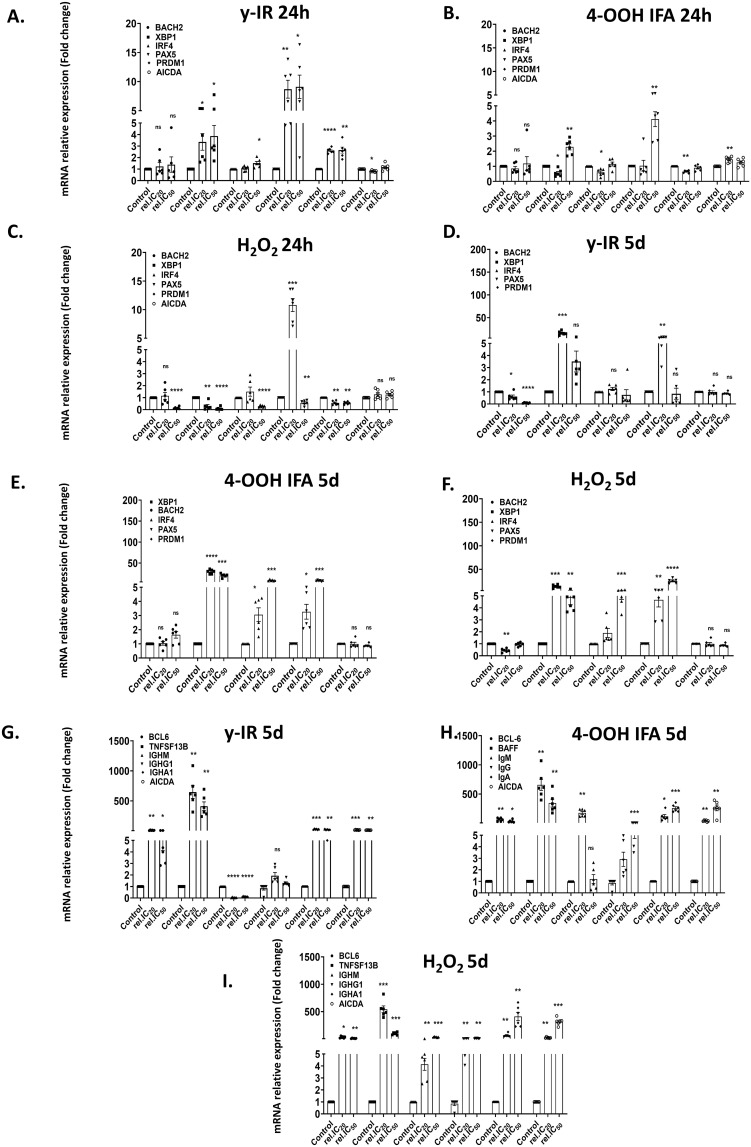
Genotoxic stress reshapes transcriptional programs of B cell differentiation, survival, and immunoglobulin lineage commitment in naive B cell-RA-FLS co-cultures. Bar charts show fold change (ΔΔCt) mRNA relative to time-matched untreated control in whole naive B cell-RA-FLS co-cultures after a single rel.IC_20_ or rel.IC_50_ dose/concentration of γ-IR **(A, D, G)**, 4-OOH IFA **(B, E, H)**, or H_2_O_2_ **(C, F, I)**, measured at 24 h or day 5. First and second rows **(A–F)** depict B cell differentiation regulators BACH2, XBP1, IRF4, PAX5, PRDM1, and AICDA at 24 h **(A-C)** and day 5 **(D–F)**; the third row **(G–I)** shows expression of BCL6, TNFSF13B, IGHM, IGHG1, IGHA1, and AICDA at day 5 following rel.IC_20_ or rel.IC_50_ exposure to each stressor. In all panels, rel. IC_20_ and rel.IC_50_ denote relative effect levels that are used as sublethal benchmarks, as defined in the Methods and **Figure 1**. All data are presented as mean ± SEM with one value per donor and dose/concentration, expressed as percentage or fold change relative to the corresponding untreated control. Group differences versus untreated control were analyzed using repeated-measures one-way ANOVA with Dunnett’s post-hoc test; *p ≤ 0.05, **p ≤ 0.01, ***p ≤ 0.001, ****p ≤ 0.0001, ns: not significant. Naive B cell-RA-FLS co-cultures: N = 6 donors, n = 1.

After 24 hours, γ-IR exposure led to coordinated upregulation of XBP1, PAX5, and PRDM1 at both IC_20_ and IC_50_, and IRF4 at IC_50_, consistent with early activation of the B cell differentiation network. In contrast, 4-OOH IFA triggered selective downregulation of XBP1, IRF4, and PRDM1at IC_20_, while PAX5 and XBP1 were upregulated only at IC_50_. H_2_O_2_ treatment induced broad repression of differentiation-associated transcripts. BACH2, XBP1, IRF4, PAX5, and PRDM1 were all downregulated at IC_50_, with PAX5 remaining the only gene elevated at IC_20_ (**Figures 4A–C**). In addition, AICDA was downregulated at γ-IR IC_20_ and upregulated at 4-OOH IFA IC_20_, with no significant modulation under the remaining conditions. Five days post-treatment, transcriptional trajectories diverged across genotoxins. For γ-IR, XBP1 and PAX5 remained upregulated at IC_20_, while BACH2 was selectively downregulated at IC_20_ and IC_50_ (**Figure 4D**). After 4-OOH IFA and H_2_O_2_ exposure, XBP1, IRF4, and PAX5 were uniformly increased at both concentration levels, except for IRF4 at IC_20_ under H_2_O_2_, which remained unchanged (**Figures 4E, F**). BACH2 was downregulated under H_2_O_2_ IC_20_. At the same late time point, transcription of germinal center and effector regulator genes was strongly induced. BCL6 was markedly upregulated, reaching 10-15-fold for γ-IR and up to 100-fold for 4-OOH IFA and H_2_O_2_ at both IC_20_ and IC_50_. TNFSF13B rose sharply to ~600-700-fold under all stressors. Among immunoglobulin heavy chains, IGHM transcripts declined in both IC_20_ and IC_50_ of γ-IR, increased in IC_20_ 4-OOH IFA, and enhanced in both IC_20_/IC_50_ for H_2_O_2_. IGHG1 transcripts were elevated only under 4-OOH IFA IC_50_ and H_2_O_2_ (both IC_20_ and IC_50_), while IGHA1 transcripts, together with AICDA, were consistently increased under all stressors at both concentrations (**Figures 4G–I**). In RA−FLS monocultures at 24 hours, the previously published study (Bruci et al., 2026) showed only modest, stimulus−limited induction of selected differentiation factors (BACH2, PAX5, IRF4, PRDM1, XBP1, and AICDA) under H_2_O_2_ and 4−OOH IFA, in contrast to the broader and temporally dynamic transcriptional patterns observed in naïve B cell-RA-FLS co-cultures under the same sublethal conditions (19).

Despite these widespread changes, no other measured transcriptional markers displayed significant modulation, indicating that the differentiation program response is selective. Because these measurements were performed on bulk co-culture samples, they do not resolve cell-type-specific regulation within naïve B cells versus RA-FLS or aggregates. Collectively, these data reveal that γ-IR, 4-OOH IFA, and H_2_O_2_ are associated with distinct temporal patterns of B cell differentiation gene expression, with both early and late changes observed in selected transcription factors and effector regulator genes. These transcriptional changes prompted us to assess whether prolonged genotoxic stress in naive B cell- RA-FLS co-culture would also be reflected at the level of cytokine secretion and B cell subset composition.

### 3.6 Prolonged treatment reduces IL-10 secretion and expands B cell-derived CD90^+^podoplanin^+^ aggregates in RA-FLS co-cultures

**Figure 5:**
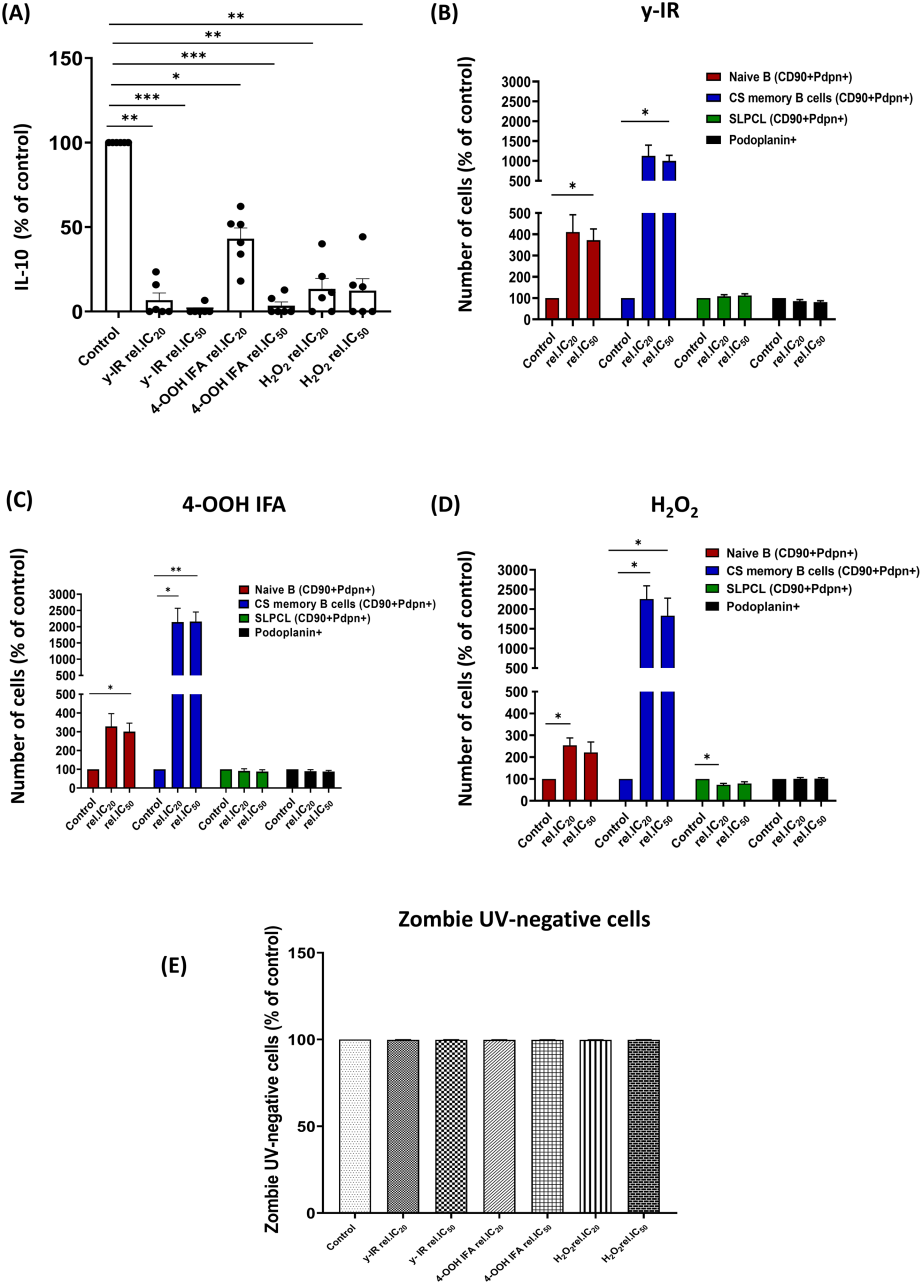
Sublethal genotoxic stress reshapes IL-10 production and B cell subset composition in naive B cell-RA-FLS co-cultures. **(A)** IL-10 levels in culture supernatants measured 8 days (day 9) after a single rel.IC_20_ or rel.IC_50_ exposure to γ-IR, 4-OOH IFA, or H_2_O_2_, expressed as percentage of the untreated control. In all panels, rel. IC_20_ and rel.IC_50_ denote relative effect levels that are used as sublethal benchmarks, as defined in the Methods. **(B–D)** Numbers of B cell subsets and podoplanin^+^ RA-FLS within naive B cell-RA-FLS co-cultures at day 9 after **(B)** γ-IR, **(C)** 4-OOH IFA or **(D)** H_2_O_2_, normalized to the corresponding untreated control, with Naive B (CD90^+^Pdpn^+^) in red, CS memory B cells (CD90^+^Pdpn^+^) in blue, SLPC (CD90^+^Pdpn^+^) in green and podoplanin^+^ RA-FLS in black. At this time point, Naive B (CD90^+^Pdpn^+^), CS memory B (CD90^+^Pdpn^+^), and SLPC (CD90^+^Pdpn^+^) denote B cell-derived CD90^+^podoplanin^+^ aggregates corresponding to naive B cells, class-switched memory B cells, and short-lived plasma-like cells, respectively, defined by their retained B cell markers. **(E)**. Percentage of Zombie UV-negative cells in naive B cell-RA-FLS co-cultures at day 9 (8 days after treatment), used as an indicator of membrane integrity within flow cytometry measurements and expressed relative to the corresponding untreated control. All data are presented as mean ± SEM, with one value per donor and dose/concentration, expressed as percentages or fold changes relative to the corresponding untreated control. Group differences versus untreated control were analyzed using repeated-measures one-way ANOVA with Dunnett’s post-hoc test; *p ≤ 0.05, **p ≤ 0.01, ***p ≤ 0.001, ****p ≤ 0.0001, ns: not significant. Naive B cell-RA-FLS co-cultures: N = 6 donors, n = 1.

To investigate the impact of prolonged genotoxic stress on B cell subset dynamics and phenotype in the naive B cells-RA-FLS co-culture system, cells were exposed to γ-IR, 4-OOH IFA, or H_2_O_2_ at IC_20_ and IC_50_ concentrations, followed by 8 days of stimulation. In the co-culture system exposed to genotoxic stress, analysis of supernatants from the whole co-culture revealed a specific cytokine response, with notable downregulation of IL-10 across all three treatments at both IC_20_ and IC_50_ concentrations (**Figure 5A**). This reduction in IL-10 was consistent in the supernatant collected after nine days of co-culture, indicating a suppressive effect of genotoxic stress on IL-10 secretion. Interestingly, other cytokines/immunoglobulins, such as CD40, IL-4, TACI, IgM, IgG, IgA, IgE, TNF, and IFN-γ, were not detectable despite being readily detectable in parallel positive control samples used to validate assay performance. The specific decrease in IL-10 suggests that genotoxic stress might impair the production of anti-inflammatory or regulatory cytokines in this co-culture environment. To explore whether these effects might be driven by population-level differences in membrane integrity, the proportion of Zombie UV-negative cells was quantified in flow cytometry measurements as an indicator of membrane integrity. Across all treatments and concentrations, the frequency of Zombie UV-negative cells remained consistently high (>97%) and did not differ significantly from untreated controls (**Figure 5E**).

Following the observed transcriptional changes in co-culture under genotoxic stress, the composition of these cell populations was further investigated. This approach aimed to clarify how genotoxic treatments influence the differentiation, distribution, and marker expression of functionally distinct B cell subsets, thereby providing additional insight into observed transcriptional profiles and cytokine/immunoglobulin secretion patterns. Prior to use in co-culture, naive B cells were sorted to >96% purity, as confirmed by flow cytometry, and were negative for both CD90 and podoplanin expression (**Supplementary Figure 9A**). Similarly, cultured RA-FLS showed no expression of CD19, CD27, or other B-cell lineage markers, confirming their distinct stromal identity (**Supplementary Figure 9B**).

In line with these baseline phenotypes, early readouts from the Ki-67 experiments already indicated that this separation between immune and stromal compartments was not stable over time, as naive B cells began to acquire CD90 expression within the first 48 hours of co-culture. During prolonged co-culture, this early convergence became even more pronounced. Notably, the podoplanin^+^ population exhibited low CD90 expression, whereas the majority of B cells across subsets demonstrated concurrent positivity for CD90 and podoplanin after 8 days post-stimulation, complicating the distinction between B cells and RA-FLS populations. CD90 and podoplanin expression became near-universal, precluding reliable discrimination of classical B cell subsets from stromal cells at this late time point, despite rigorous gating of singlet events using FSC-A vs. FSC-H to exclude doublets and aggregates (**Supplementary Figure 5**). Multiple attempts to improve dissociation, including Accutase, Accutase with 4 mM EDTA, cold PBS, citrate buffer, dispase combined with Liberase and DNase, and Liberase alone or in various combinations, failed to re-establish clearly separated populations (**Suplementary Figure 9C**). For these dissociation experiments, SSC−A versus SSC−H pulse geometries alongside FSC−A versus FSC−H were examined and found no significant change in the proportion of doublets or aggregates compared with the main FSC−based singlet gate (**Supplementary Figure 11**). In parallel, naive B cells were pre-labeled with CellTrace CFSE to support lineage tracking in co-culture, but by day 9, CFSE fluorescence was uniformly detected across the culture, precluding its use for reliable discrimination of B cell and RA-FLS compartments.

By day 9, extensive co-expression of CD90 and podoplanin was therefore observed across B cells and RA-FLS, indicating stable physical interactions and phenotypic convergence in co-culture, without redistribution of B cell markers such as CD19 or CD27 onto RA-FLS. This was further supported by unstained and isotype controls, as well as RA-FLS monocultures, which showed no expression of B cell markers (**Supplementary Figure 9B**). In light of this, the populations referred to as naive B cells, class-switched memory B cells, and short-lived plasma-like cells at this late time point are best understood as B cell-derived CD90^+^podoplanin^+^ aggregates defined by their retained B cell markers, and these labels are used as operational terms for readability and continuity with earlier time points.

Within this aggregated phenotype, genotoxic treatments induced distinct changes in B-cell-derived subsets. Following γ-IR, upregulation of the naive B cell and class-switched memory B cell-aggregate compartments was observed at IC_50_. Short-lived plasma-like cell aggregates and podoplanin^+^ populations remained unchanged (**Figure 5B**). Comparable patterns were detected after 4-OOH IFA treatment: naive B cell aggregates were selectively enriched at IC_50_ and class-switched memory B cell aggregates at both IC_20_ and IC_50_, with no effect on short-lived plasma-like cell aggregates or podoplanin^+^ cells (**Figure 5C**). H_2_O_2_ exposure resulted in upregulation of class-switched memory B cell aggregates at both concentrations and naive B cell aggregates only at IC_20_, accompanied by a decrease in short-lived plasma-like cell aggregates at IC_20_ (**Figure 5D**). Furthermore, short-lived plasma-like cell aggregates displayed the highest baseline podoplanin MFI among the populations, whereas CD90 baseline MFI was not significantly different across groups. In direct comparison within each cell subset, podoplanin MFI was consistently greater than CD90 MFI (**Supplementary Figures 10A, B**).

Among naive B cells aggregates in co-culture, the MFI of podoplanin decreased following H_2_O_2_ and 4-OOH IFA treatment. In class-switched memory B cell aggregates, podoplanin MFI declined only with γ-IR treatment, whereas short-lived plasma-like cell aggregates showed increased podoplanin MFI for all three genotoxic exposures. Similar trends were observed for CD90 MFI: naive B cells and class-switched memory B cells aggregates presented a reduction of CD90 MFI after all three treatments, and short-lived plasma-like cells aggregates showed an increase of CD90 MFI after all three treatments (**Supplementary Figures 10C, D**). These findings collectively highlight that prolonged genotoxic stress induces selective expansion and phenotypic remodeling of specific B cell subsets within the RA-FLS co-culture, accompanied by a consistent downregulation of the anti-inflammatory cytokine IL-10, suggesting a complex interplay between DNA damage, cellular phenotype, and immune regulatory functions that warrants further investigation.

## 4 Discussion

Our data reveal differential sensitivity to genotoxic stress among RA-FLS, naive B cells, and memory B cells, as measured by the Annexin V/PI assay. In naive B cell-RA-FLS co-cultures, we observed selective transcriptional activation of DNA damage response genes. The observed preferential sensitivity of memory B cells compared with naive B cells and RA-FLS is consistent with reports that lymphocyte subsets, including memory populations, exhibit distinct DNA damage responses, suggesting potential differences in stress tolerance across compartments (22). The selective activation of core DNA damage sensors and apoptotic mediators might support the engagement of canonical DNA damage and stress pathways rather than a broad effector response at early timepoints in this system (23, 24).

The robust upregulation of ATM, APEX1, and RAD50 at high dose/concentration levels might be related to double-strand break/base excision repair machinery, known as critical early responders to genotoxic injury (24, 25). However, direct measurement of repair pathway activity was not performed in this study, and these interpretations remain inferential. The induction of BAX in response to irradiation and hydrogen peroxide, but not alkylating agents, may reflect context-dependent apoptotic regulation, consistent with reports that distinct genotoxic insults can differentially modulate stress signaling; the specific apoptosis pathways engaged in B cells remain to be defined (26, 27). BCL6 upregulation following irradiation may be associated with roles in regulating cell survival and apoptosis among B cell subsets, as previously reported (43).

The comparative resistance of RA-FLS to cytotoxic stress may relate to their established resilience in inflamed synovial environments, possibly mediated by upregulation of survival pathways or antioxidant defenses (28). It is conceivable that these cells might possess transcriptional or post-translational mechanisms buffering against stress-induced apoptosis, but this was not directly addressed in this study. Notably, the absence of transcriptional modulation in several other DNA damage and effector genes might suggest a limited breadth of early response, with only a subset of sensors and mediators activated under acute stress (29). This pattern may reflect stimulus type, concentration, or temporal kinetics.

### 4.1 γ-H2AX signal patterns, cell-cycle control, and lineage context

Building upon our initial findings of differential stress sensitivity and selective DNA damage response gene activation in RA-FLS and naive B cell co-culture, time course analysis of γ-H2AX MFI highlights further lineage-specific distinctions in damage signaling patterns. Notably, RA-FLS exhibit intrinsically higher proliferative rates than quiescent naive B cells, which influences their cell cycle distribution and repair context (30). In the co-culture setting, however, naive B cells upregulated CD90 within 48 hours and subsequently formed stable CD90^+^podoplanin^+^ aggregates with RA-FLS, indicating close physical interactions and a shared microenvironment rather than strictly isolated lineages. Accordingly, at later time points, populations referred to as naive B cells actually represent B cell-enriched, CD90^+^podoplanin^+^ aggregates rather than fully pure naive B cells. Within this context, γ-H2AX MFI increased in B cell enriched aggregates at earlier time points and then decreased over the 0–24 h observation window, describing a characteristic γ-H2AX signal pattern in the B cell derived compartment. This profile resembles published observations of dynamic γ-H2AX turnover in human blood cells (22, 31, 32). However, in our setting it should be interpreted purely as a descriptive signal pattern, not as evidence for specific rates or mechanisms of DNA repair, which were not measured. By contrast, RA-FLS showed higher γ-H2AX MFI at later time points within the same window, in line with reports that synovial fibroblasts can mount strong DNA damage responses and show prolonged checkpoint activation after genotoxic stress (33). The persistently higher baseline γ-H2AX signal in RA-FLS may reflect chronic exposure to oxidative stress or ongoing low-grade DNA damage characteristic of the inflamed synovial environment, but within our experiments it primarily indicates a higher steady state level of damage-associated signaling rather than a quantified burden of DNA lesions (8, 34).​ Integrating γ-H2AX profiles with cell cycle analyses, both naive B cell-enriched aggregates and RA-FLS initially exhibited an enrichment in the late S- early G_2_-M phases following genotoxic exposure, a pattern that might be compatible with checkpoint engagement in response to DNA damage as observed in other systems (33, 35). Nonetheless, because γ-H2AX MFI was assessed at discrete time points without direct measurement of DNA strand breaks, repair intermediates, or pathway activity, the available data do not allow conclusions about the rate or efficiency of DNA repair in either lineage. Accordingly, these combined γ-H2AX and cell-cycle readouts are interpreted as evidence of distinct lineage- and context-dependent responses to genotoxic stress, rather than as a quantitative comparison of repair kinetics between B cells and RA-FLS. Given the early acquisition of CD90 and aggregate formation, these patterns reflect responses of B cell enriched compartments within a tightly adherent co-culture rather than completely segregated cell types. Our analysis of Ki-67^-^ populations revealed uniformly elevated G_0_ + arrested cell proportions at 48 h across all conditions, with no treatment-specific alterations. Notably, even in γ-IR experiments where only naive B cells were directly irradiated, RA-FLS displayed similar dynamics in the non-cycling compartment, suggesting that fluctuations in G_0_ + arrested cells may partly reflect co-culture interactions and bystander effects rather than cell intrinsic responses. This observation emphasizes the influence of paracrine and contact-dependent signaling in the RA-FLS/B cell co-culture system and indicates that genotoxic treatment effects are more clearly resolved within the proliferative (Ki-67^+^) fractions (36). At later time points, only RA-FLS maintained altered cell-cycle distributions, particularly at higher stressor concentrations. This persistent enrichment of late S-early G_2_/M phases in RA-FLS, together with sustained γ-H2AX signal and their proliferative drive, is compatible with the idea that RA-FLS remain in a perturbed cycling state for longer than B cell-enriched aggregates under genotoxic stress, in line with reports of increased DNA damage and altered repair in FLS (37, 38). These findings complement the observation of selective activation of DNA damage related genes and a limited breadth of early transcriptional responses, and together support the view that lineage-intrinsic properties such as proliferation rate, damage signaling, and cell-cycle control may contribute to shaping cellular adaptation to genotoxic stress, although the underlying mechanisms were not directly dissected in this study.

### 4.2 B cell fate regulators, immunoglobulin programs, and late effector blockade

The present results demonstrate that genotoxic stress elicits highly selective, time-resolved changes in expression of B cell differentiation genes within naive B cell/RA-FLS co-cultures. Exposure to γ-IR, 4-OOH IFA, and H_2_O_2_ at defined doses/concentrations altered major regulators of plasma cell and germinal center fates, as well as immunoglobulin heavy chain genes. The selective regulation of XBP1, PRDM1, PAX5, BACH2, IRF4, and BCL6 is consistent with their established roles in germinal center and plasma cell fate control (39–42). Activation of the DNA damage response and checkpoint signaling in B cells may coordinate with differentiation-associated machineries (43, 44). The transcriptional dynamics observed at both early and late time points are compatible with distinct, context-dependent gene-regulatory outcomes previously described for B cells subjected to genotoxic or metabolic stress (44). Notably, the broad upregulation of TNFSF13B (BAFF) and AICDA across all stressors may indicates engagement of transcriptional programs associated with immunoglobulin class switching and B cell effector activity (45–47).​ Elevated BAFF has been linked to relaxed peripheral B cell tolerance and increased survival of autoreactive B cells in other systems (47, 48). In the present co-culture system, however, we only demonstrate increased BAFF mRNA under genotoxic stress and do not directly assess autoreactivity or clonal persistence. Changes in immunoglobulin heavy chain transcripts, including altered IGHM, IGHG1, and IGHA1 patterns, support the notion that stress signaling influences effector differentiation stages. Reports show AID-induced genotoxic stress from DSBs activates ATM signaling to promote plasma cell differentiation despite genomic remodeling. AICDA induction is compatible with priming for class-switch recombination (CSR), yet analysis of day 9 co-culture supernatants and B cell phenotypes revealed dissociation between differentiation markers and functional output, which could reflect DDR checkpoint constraints on late secretion or proliferation but may also be influenced by the limited stimulation conditions used here (CpG plus anti-IgM without additional T cell derived signals) (43, 49, 50). In our system, this dissociation is reflected by the fact that only class-switched memory B cells expanded over time, whereas short-lived plasma-like cell aggregates numbers remain unchanged, and no IgM, IgG, IgA, or IgE, nor additional cytokines, were detectable in supernatants from either control or genotoxin-treated cultures. This pattern may indicate that the naive B cell-RA-FLS co-culture supports differentiation up to memory and short-lived plasma-like cell stages without promoting robust antibody secretion, and that genotoxic stress may contribute to maintaining a late effector bottleneck rather than driving immunoglobulin release under these conditions. Flow cytometry showed short-lived plasma-like cell aggregate formation and expansion of naive and class-switched memory B cell aggregate compartments under genotoxic stress, whereas ELISA detected no IgM, IgG, IgA, or IgE and revealed a selective reduction of IL-10, with other cytokines remaining undetectable. This combination of transcriptional priming, phenotypic differentiation, and absent secretory activity, in the context of submaximal polyclonal stimulation, is compatible with the requirement for a dedicated plasma cell secretory/UPR program for full effector function such as Blimp-1/XBP1-driven UPR, and suggests that such programs are not fully engaged in this model (43, 51, 52). Likewise, while these data reveal marked differences in gene expression dynamics between genotoxins and concentrations, the underlying mechanisms, including any roles of checkpoint-mediated inhibition of secretory pathways or RA-FLS-dependent modulation of late effector functions, remain speculative and were not directly tested here, and will require targeted mechanistic studies in more reductionist systems.

### 4.3 Phenotypic plasticity, stromal marker convergence, and microenvironmental reprogramming

Our gating strategy distinguished naive B cells, class-switched memory B cells, short-lived plasma-like cells, and podoplanin^+^ cells (CD38^-^IgG^-^CD19^-^CD27^-^podoplanin^+^). At 24 h, including all γ-H2AX analyses, B cell and RA-FLS subsets remained clearly separable, and widespread co-expression of CD90 and podoplanin was not yet detected. While naive B cells initially expressed no CD90, by day 9, we observed near-universal co-expression of CD90 and podoplanin across B cell and RA-FLS populations. This convergence complicated subset discrimination and precluded unequivocal assignment of classical immune or stromal identities at late timepoints. Accordingly, at these late timepoints, the populations referred to as naive B cells, class-switched memory B cells, and short-lived plasma-like cells represent B cell-derived, CD90^+^podoplanin^+^ aggregates defined by their retained B cell markers, rather than strictly isolated, lineage-pure subsets. Importantly, lineage origin was defined at co-culture initiation using magnetically isolated naïve B cells of >96% purity (by flow cytometry) and RA−FLS monocultures lacking B cell lineage markers. γ-H2AX and Annexin V/PI readouts were acquired within CD19^+^CD27^-^/CD19^+^CD27^+^ B cell gates or CD90^+^ RA-FLS gates, as detailed in the flow cytometry methods and **Supplementary Figures 2, 3**. For Ki-67 and phenotypic analyses at 48 h and 72 h, gating was likewise performed on CD19^+^CD27^-^/CD19^+^CD27^+^ B cell and CD90^+^ RA-FLS compartments, but these measurements increasingly reflected B cell enriched CD90^+^ (and later CD90^+^podoplanin^+^) aggregates within the tightly adherent co-culture rather than completely isolated B cells or RA-FLS. Thus, late co-expression of CD90 and podoplanin may reflect phenotypic convergence of B cell derived and stromal populations rather than uncertainty about their cellular origin. Genotoxic treatments selectively expanded naive and class-switched memory B cell aggregates, whereas short-lived plasma-like cell aggregates and podoplanin^+^ populations remained largely unchanged; however, similar patterns in untreated controls may suggest that phenotypic adaptation is not unique to genotoxic damage but also arises as an intrinsic feature of chronic co-culture. Such extensive phenotypic plasticity and marker convergence are compatible with microenvironment-driven transcriptional adaptations in addition to DNA damage-induced changes (30). In other models of chronic inflammation and tissue explants, sustained inflammatory cues induce non-canonical marker expression in lymphocytes, underscoring the role of stromal and cytokine context in shaping immune phenotypes (8, 53, 54).

In this context, the widespread acquisition of stromal markers such as CD90 and podoplanin by B cells, markers traditionally restricted to mesenchymal or fibroblastic lineages, raises questions about the underlying mechanisms. Persistent physical contact and reciprocal signaling within the co-culture environment could possibly promote transcriptional reprogramming and atypical marker upregulation (55). Additionally, membrane transfer processes like trogocytosis might contribute to the dual expression of these markers; immune cells and stromal cells are known to exchange membrane fragments during cell-cell contact (55, 56). Although we did not directly examine trogocytosis, the inability to dissociate the populations further might support the likelihood of robust physical adhesion and, potentially, more complex interaction phenomena. Such phenomena have been described in models of chronic inflammation, where persistent stromal and cytokine cues foster cellular phenotypic convergence and membrane exchange, including trogocytosis, within tightly interacting immune stromal niches (56).

The discrepancy between protein and mRNA detection of podoplanin and CD90 in B cell subsets described by Zhang et al. provides an important contextual parallel, suggesting that protein-level marker acquisition on B cells in inflamed environments may not always be accompanied by corresponding transcriptional signatures, possibly reflecting post-transcriptional regulation or uptake from neighboring stromal cells (54). This resonates with our findings, where B cells in co-culture acquire stromal markers at the protein level without clear evidence, at the bulk co-culture level, of intrinsic transcriptional induction in B cells. Together, these observations do not allow mechanistic discrimination but are compatible with a model in which strong physical interactions, microenvironmental cues, and non-canonical marker transfer could cooperate to generate atypical B cell phenotypes under prolonged genotoxic stress and chronic co-culture, and they motivate future, dedicated studies to distinguish between transcriptional reprogramming and membrane transfer.

### 4.4 Cytokine modulation and potential functional skewing

Genotoxic stress may further modulate these processes by activating DDR and constraining effector programs, potentially favoring survival/expansion over terminal effector maturation. This is compatible with sustained induction of DDR genes (ATM, RAD50, APEX1) together with differentiation regulators (PRDM1, IRF4), and with evidence that ATM/DDR signaling can shape differentiation outcomes in a context-dependent manner (43, 44, 57). The cytokine profile corroborates these phenotypic observations, showing near-complete suppression of IL-10 secretion across all genotoxic conditions. Given that IL-10 production is controlled by defined transcriptional programs (including STAT3-linked circuits in IL-10 producing B-cell contexts), altered engagement of these regulatory pathways could contribute to reduced IL-10 output, although this was not directly tested here (58). In the absence of broader functional assays (for example on T cells or additional regulatory cytokines), the observed IL-10 reduction is best interpreted as an observed reduction in a potential regulatory cytokine signal within the co-culture, and any consequences for IL-10-mediated negative feedback remain to be determined (59).

### 4.5 Integrated limitations and future directions

While this study provides new insights into B cell-stromal interactions and immune adaptation under prolonged genotoxic stress, its focused design also entails several limitations. Firstly, only naive B cells were investigated, and monocultures rapidly lost viability and could not be maintained beyond 48 hours, which necessitated the use of an RA-FLS-supported co-culture system to enable long-term kinetic and functional analyses. This dependence on stromal support constrains purely B-cell-intrinsic interpretation but, at the same time, allows DNA damage responses and fate decisions to be interrogated in a more pathophysiologically relevant niche. In addition, data for RA-FLS cultured alone, including RT-qPCR-based characterization of their stress and inflammatory programs under sublethal genotoxic conditions, have been reported previously (Bruci et al., 2026) and were not repeated in full here; instead, the present study was deliberately designed to focus on the interactive context, with RA-FLS used as a well-defined stromal comparator rather than having their baseline phenotype re-established (19). Pronounced phenotypic convergence and technical challenges in dissociating large aggregates limited fully resolved lineage discrimination at late time points, yet these same features indicate that the model captures intense, chronic cell-cell interactions that are difficult to achieve in simpler systems. Because late co-cultures formed tight B cell-RA-FLS aggregates that could not be fully dissociated, flow-cytometric analyses were performed using conservative FSC-A versus FSC-H singlet gating, and late readouts are therefore best interpreted as properties of B cell-derived aggregates rather than fully isolated single cells. Furthermore, transcriptional profiling of co-cultures at early and late time points was carried out on bulk RNA, so cell-type-specific gene regulation within individual B cell subsets or RA-FLS populations could not be resolved and will require future single-cell or sorted-population approaches. In line with this, dedicated transcriptional measurements of stromal markers such as THY1/CD90 and PDPN were not included in the 24-hour RT-qPCR panel, and THY1/PDPN transcript dynamics could therefore not be assessed in a compartment-resolved manner. Protein-level analyses indicated constitutive expression of CD90 and podoplanin in RA-FLS and relatively stable podoplanin mean fluorescence intensity in the stromal compartment across genotoxic conditions, whereas the most pronounced phenotypic changes were observed in B-cell-derived aggregates. Nonetheless, contributions from stromal remodeling or mesenchymal−like reprogramming to the bulk transcriptional profiles at later time points cannot be fully excluded, and compartment−resolved analyses of stromal marker expression in purified cell populations, potentially in combination with high−resolution imaging of CD90/podoplanin localization, are therefore identified as an important goal for future work. Direct quantification of DNA lesion spectra, repair pathway activity, and ATM/ATR dependence at the single-cell level was beyond the scope of this work, so DNA damage responses are inferred from γ-H2AX signal intensity, cell-cycle distribution, and targeted transcript profiling rather than from direct measurements of double-strand break repair. Taken together, these constraints define a focused, niche-centered framework that now provides a foundation for future studies incorporating additional B cell subsets, patient-derived B cells, non-RA fibroblasts, and higher-resolution single-cell and mechanistic approaches. Moreover, osteoarthritis-derived fibroblast-like synoviocytes (OA-FLS) or other non-RA fibroblasts were not included as disease controls, which precludes direct comparison of stress responses between inflammatory and less inflamed synovial fibroblast populations, but clearly positions RA-FLS as a disease-relevant stromal partner for subsequent comparative work. Finally, multiple comparisons were controlled for within each predefined endpoint using Dunnett’s test, and results are interpreted with a focus on consistent, biologically coherent patterns rather than isolated marginal effects, without applying an additional global adjustment across all readouts.

**Figure 6:**
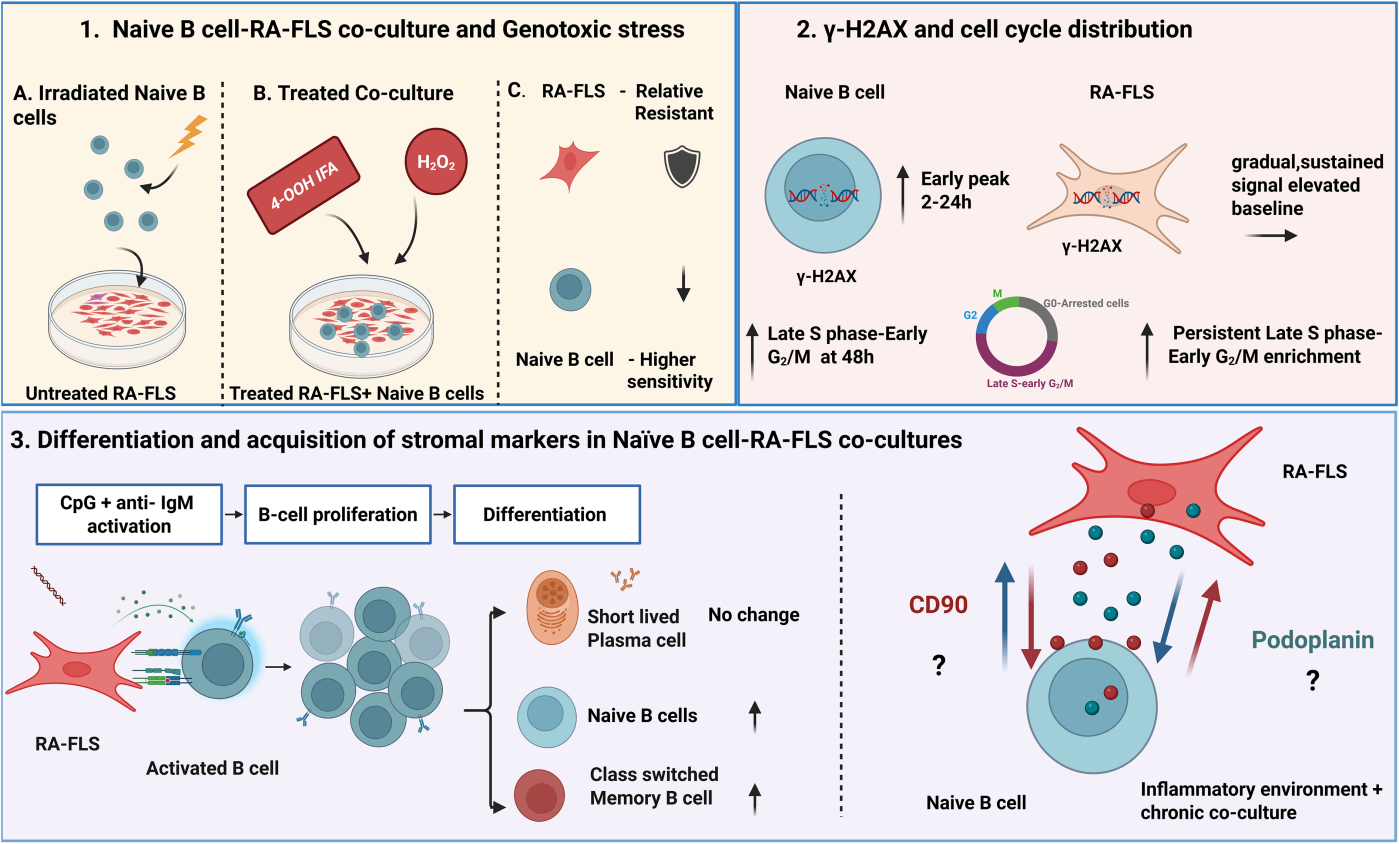
Graphical summary of naive B cell-RA-FLS co-culture responses to genotoxic stress. Panel 1 illustrates the experimental setup, where naive B cells are either γ-irradiated before addition to untreated RA-FLS (left) or naive B cell-RA-FLS co-cultures are directly exposed to 4-hydroperoxyifosfamide (4-OOH IFA) or hydrogen peroxide (H_2_O_2_) at defined relative IC_20_/IC_50_ concentrations (middle), resulting in higher stress sensitivity of naive B cells and relative resistance of RA-FLS (right). Panel 2 summarizes lineage-specific γ-H2AX mean fluorescence intensity (MFI) and cell-cycle profiles: naive B cells show a higher γ-H2AX peak between 2–24 h with subsequent attenuation, whereas RA-FLS display a more gradual but sustained γ-H2AX signal with an elevated baseline; both lineages exhibit increased late S-early G_2_/M at 48 h, with persistent late S-early G_2_/M enrichment observed only in RA-FLS at later time points. Panel 3 depicts differentiation outcomes and stromal marker acquisition in naive B cell-RA-FLS co-cultures under genotoxic stress, in which CpG + α-IgM activated B cell-derived CD90^+^podoplanin^+^ aggregates retain naive, class-switched memory, and short-lived plasma-like cell marker patterns, and chronic inflammatory co-culture is associated with increased CD90 and podoplanin expression on these B cell-derived populations alongside RA-FLS, indicating phenotypic adaptation and stromal marker convergence without assigning a specific mechanism. Image 6 was created using BioRender, used with permission.

Together, these findings suggest that genotoxic stress elicits distinct, genotype- and concentration-specific transcriptional responses in B cells within an inflammatory microenvironment. These relationships are summarized in a graphical overview that combines the experimental design, lineage-specific DNA damage responses, and differentiation outcomes in the naive B cell-RA-FLS co-culture (**Figure 6**). The selectivity of these transcriptional changes, alongside the absence of widespread transcriptomic alterations in unrelated markers, points to a focused regulatory program rather than generalized transcriptional upheaval (44). Future investigations will be required to determine how these transcriptional programs integrate at the functional level and whether they represent adaptive, maladaptive, or context-dependent outcomes of DNA damage in B cell fate regulation. Collectively, the data identify prolonged, sublethal genotoxic stress as a potent modulator of B cell differentiation and effector programs in an RA-like stromal niche and provide a conceptual framework for dissecting how DNA damage interfaces with autoimmune B cell responses.

## 5 Conclusion

This study describes distinct responses of rheumatoid arthritis fibroblast-like synoviocytes (RA-FLS) and B cell-enriched compartments derived from naive B cells under genotoxic stress, including differences in viability, γ-H2AX signal intensity, transcriptional modulation of DNA damage and differentiation-associated genes, and phenotypic changes during prolonged co-culture. The acquisition of stromal markers by B cells within the co-culture and the reduction of IL-10 production indicate cellular adaptations within an inflammatory microenvironment that may influence local immune regulation. These observations add to current knowledge of cellular interactions that are relevant to the rheumatoid arthritis synovium, where RA-FLS contribute to chronic inflammation and tissue damage and B cells participate in local immune processes. Understanding these dynamics might be clinically relevant, as could help to inform targeted strategies to modulate synovial cell behavior and immune responses, thereby improving therapeutic approaches for rheumatoid arthritis by explicitly addressing the interplay between immune and stromal cells in the joint microenvironment.

## Data Availability

The raw data supporting the conclusions of this article will be made available by the authors, without undue reservation.
