# Supplementary material for: Genotoxic stressors mimicking synovial microenvironment modulate B cell fate in RA-FLS co-culture
Source: Front Immunol. 2026 Jul 13;17:1892572. doi: 10.3389/fimmu.2026.1892572 (PMC13402539; doi:10.3389/fimmu.2026.1892572)
Supplement: Supplementary file 1 [file DataSheet1.docx]

Supplementary Material

# Supplementary Figures and Tables

## Supplementary Figures

**
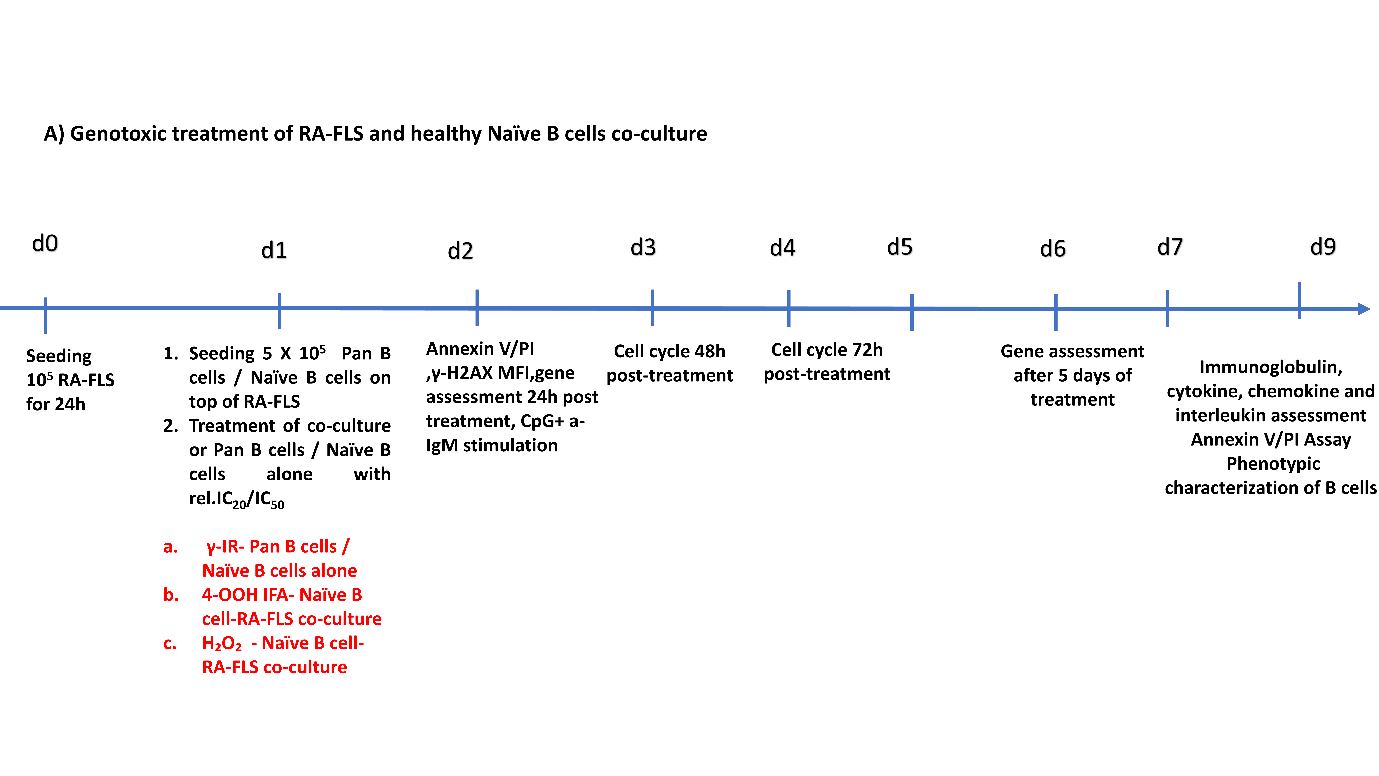
**

**Supplementary Figure 1. Overview of the naive B cell-RA-FLS genotoxic stress workflow.** RA‑FLS were plated one day before treatment (day -1), and naive B cells were introduced on day 0 to establish co-cultures that then received γ‑IR, 4‑OOH IFA, or H₂O₂ at relative IC₂₀/IC₅₀ levels. γ‑IR was applied ex vivo to naive B cells prior to co-culture, whereas 4‑OOH IFA and H₂O₂ were added directly to the co-cultures. The schematic summarizes the timing of all downstream read‑outs, including early γ‑H2AX flow cytometry (0-24 h), 24‑h viability and RT‑qPCR panels, Ki‑67/PI‑based cell‑cycle profiling at 48 h and 72 h, a delayed RT‑qPCR harvest on day 6 (5 days post treatment), and day 9 supernatant collection for ELISA and terminal B cell phenotyping. All datasets were normalized to time-matched untreated controls; full experimental details (concentrations, replicate structure, and statistics) are provided in Materials and Methods.

**
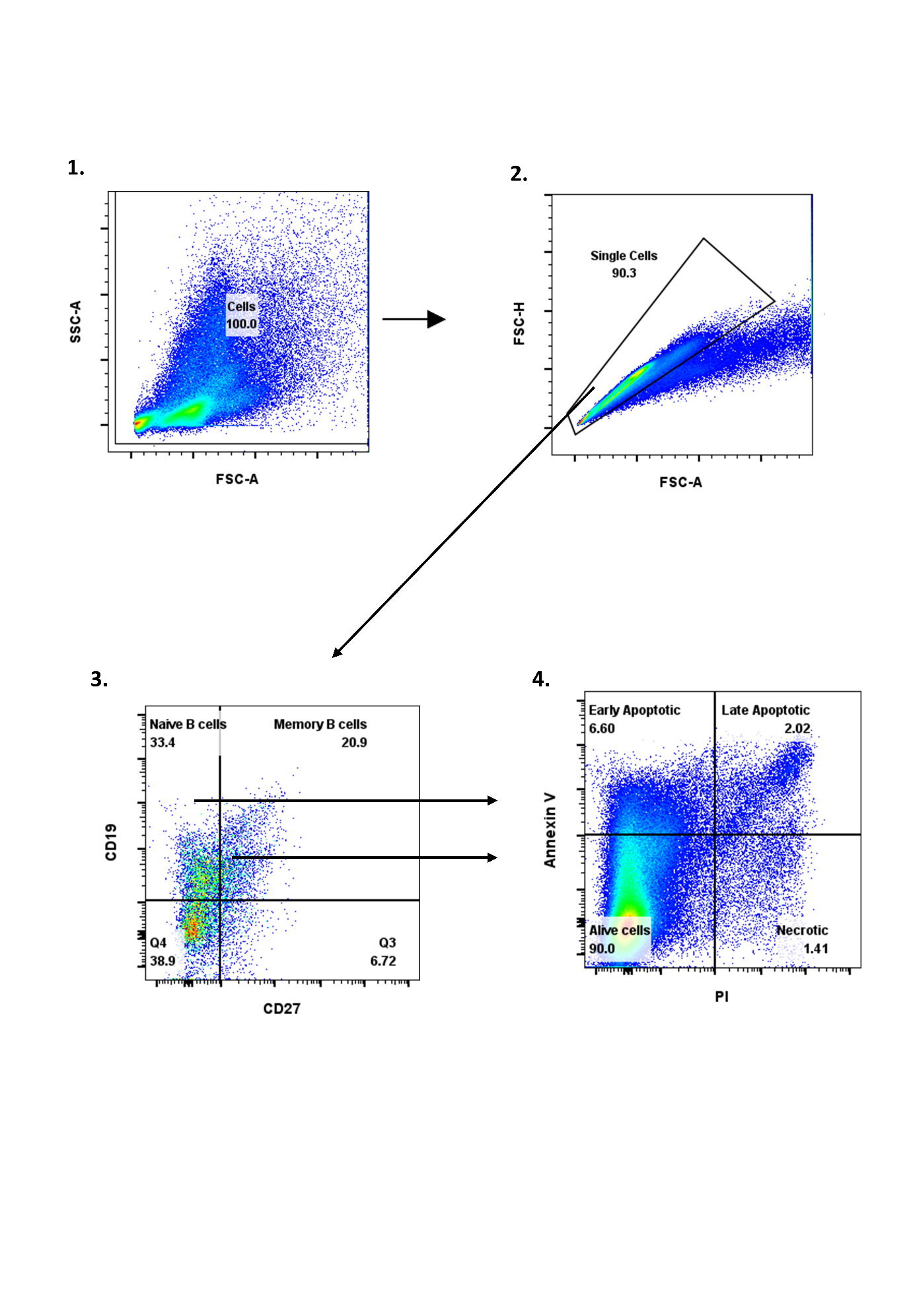
**

**Supplementary Figure 2. Representative flow cytometric gating scheme for the viability assay (illustrative, without statistical evaluation).** Total leukocytes were first defined on the FSC-A versus SSC-A plot, excluding debris (1). Single-cell events were then selected using an FSC-H versus FSC-A gate (2). CD19⁺ B cells were identified and further subdivided into naive and memory subsets based on CD27 expression (3). Finally, Annexin V-FITC and PI staining were used to delineate viable (Annexin V⁻/PI⁻), early apoptotic (Annexin V⁺/PI⁻), late apoptotic (Annexin V⁺/PI⁺), and necrotic (Annexin V⁻/PI⁺) cells in RA-FLS/healthy B cells co-cultures, with the viable fraction (Annexin V⁻/PI⁻) providing the percentages shown in Figure 1 (4).


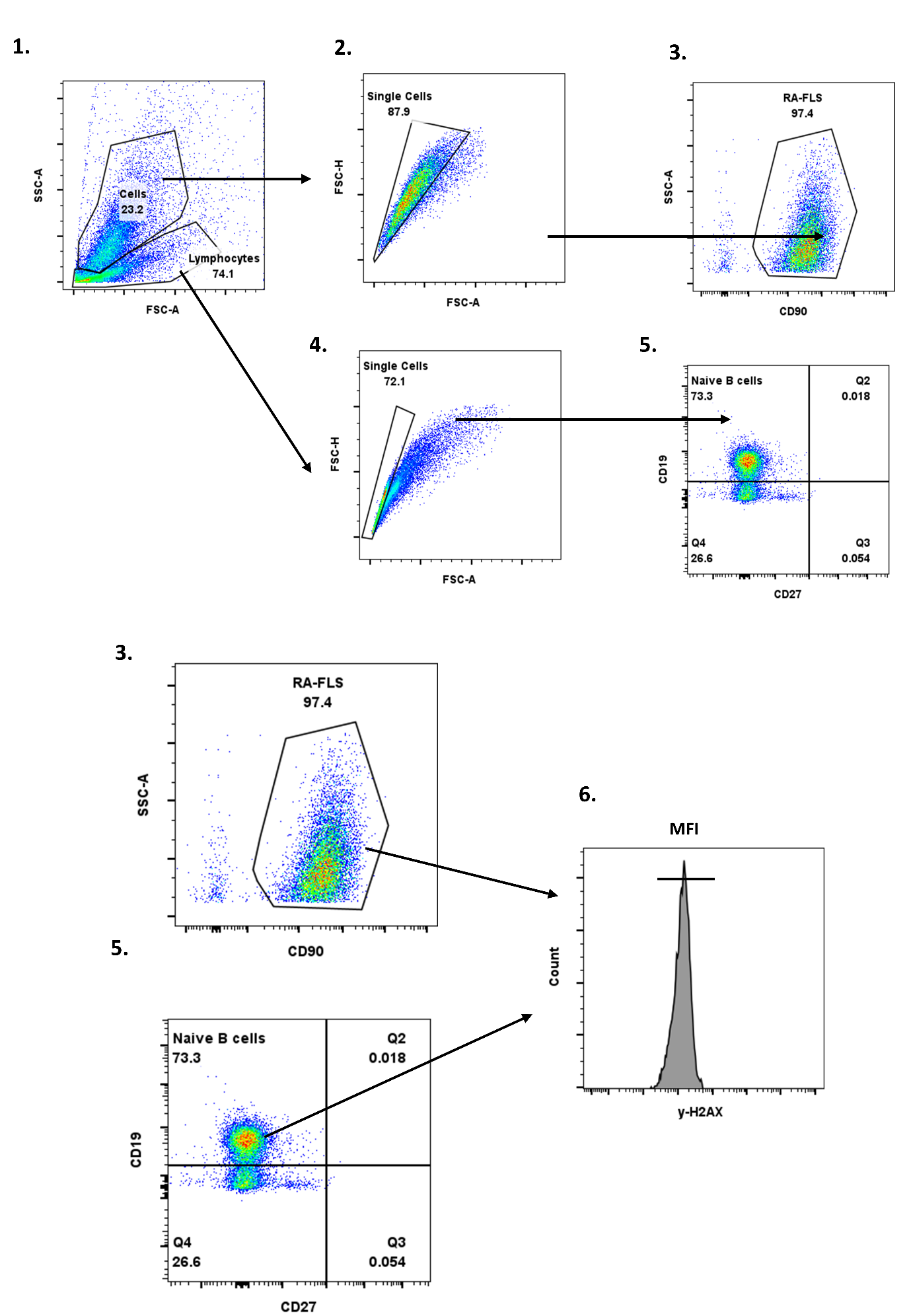


**Supplementary Figure 3. Representative flow cytometric gating workflow for the γ-H2AX assay in naive B cell / RA-FLS co-cultures.** Events were first displayed on an FSC-A versus SSC-A plot to distinguish two populations: lymphocytes (lower population) and RA-FLS (upper population characterized by larger size and lower frequency, consistent with the 1:5 RA-FLS: naive B cell ratio (1). Both populations were independently subjected to FSC-H versus FSC-A doublet exclusion gating (2,4). The single cells were further analyzed: RA-FLS were identified as CD90⁺ (3), while B cells were subdivided into naive B cells (CD19⁺ CD27⁻) (5). Finally, the median fluorescence intensity (MFI) of γ-H2AX-Alexa Fluor 488 was assessed by histogram analysis for each population under the respective treatment conditions and time points (6).


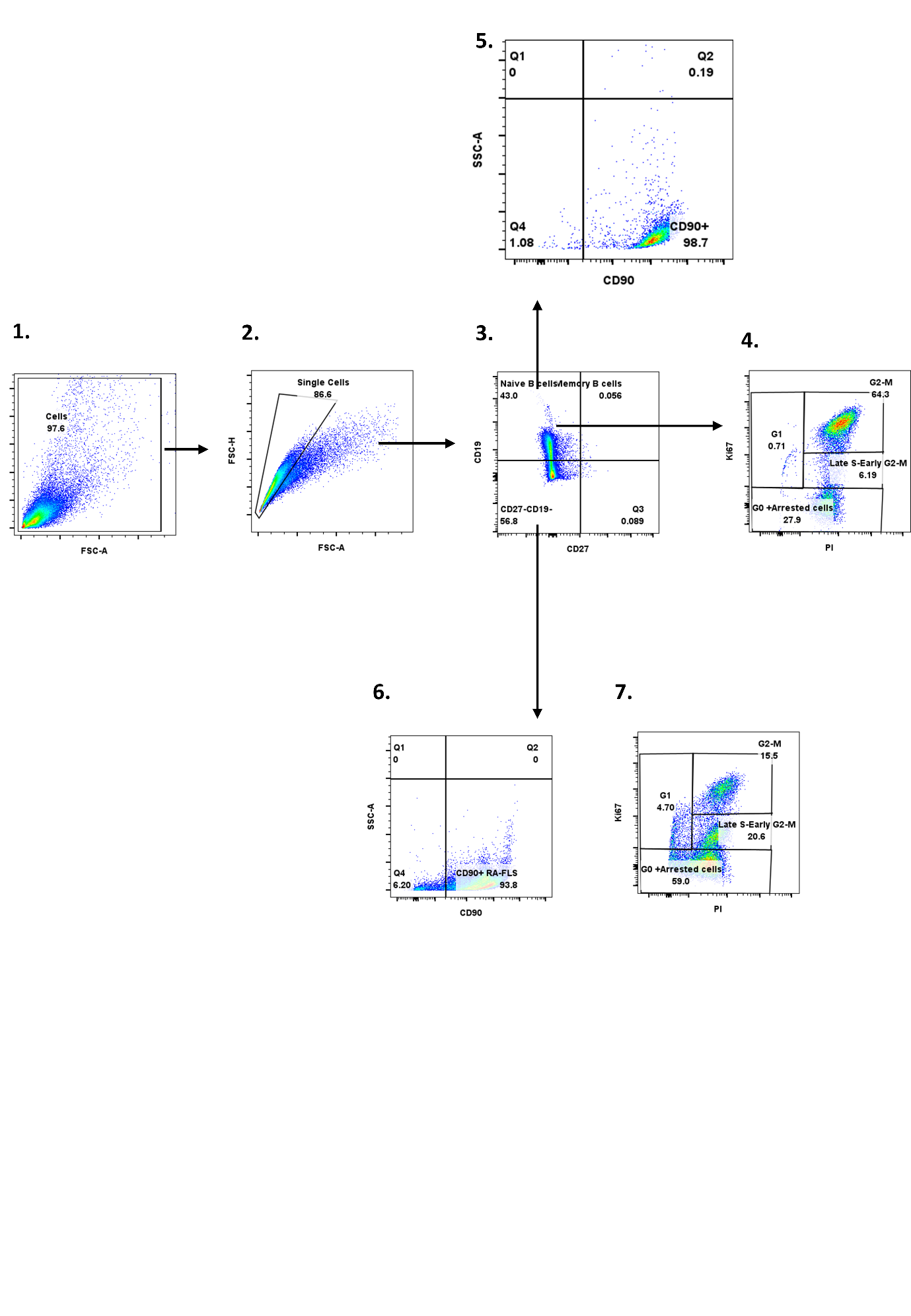


**Supplementary Figure 4. Representative gating strategy for Ki-67-FITC/propidium iodide (PI) cell cycle analysis in RA-FLS/naive B cell co-cultures**. The total cell population was first defined on the FSC-A versus SSC-A plot (1), followed by singlet discrimination using FSC-H versus FSC-A gating (2). Single cells were then resolved into naive B cells (CD19⁺ CD27⁻) and memory B cells (CD19⁺ CD27⁺) in the upper panel (3).CD19-CD27- cells were gated into CD90⁺ RA-FLS in the lower panel using SSC-A versus CD90 gating (4).  At 48 h and 72 h, events within the CD19⁺CD27⁻ gate had uniformly acquired CD90 expression, reflecting the formation of B cell-derived CD19⁺CD27⁻CD90⁺ naive B cell-stromal aggregates in co-culture (5). Cell cycle distribution was subsequently analyzed for each population using Ki-67 versus PI bivariate plots, with gates delineating G₀ + Arrested cells (Ki-67^-^/PI^+^), G₁ (Ki-67⁺/PI^-^), late S-phase/early G₂-M (Ki-67⁺/PI intermediate), and G₂-M (Ki-67⁺/PI^+^) (6,7). Identical gating templates were consistently applied across all experimental conditions and time points, with data acquired on a CytoFLEX LX flow cytometer (Beckman Coulter) and analyzed using FlowJo v10.8.1


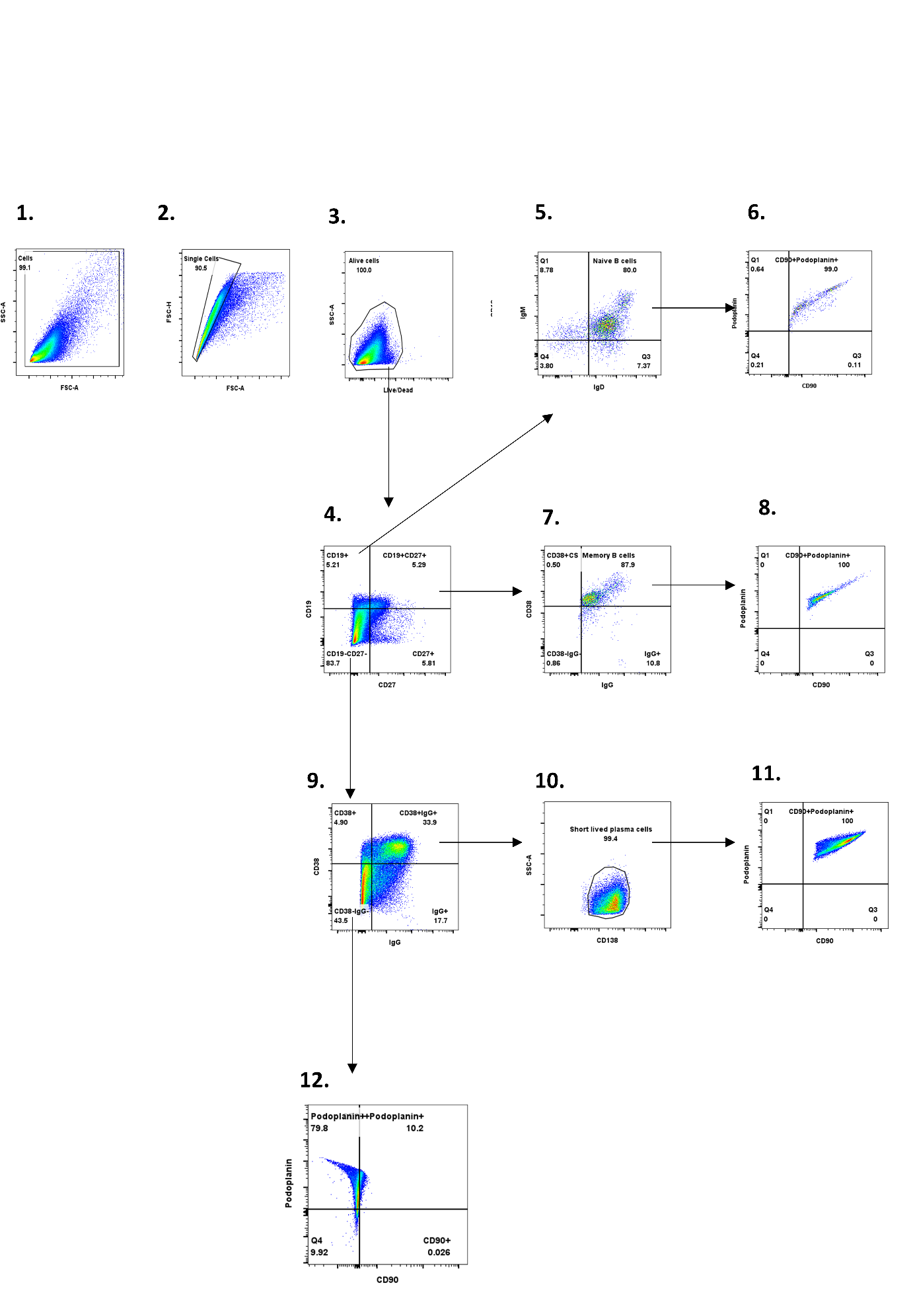


**Supplementary Figure 5.  Representative flow cytometric gating strategy for B cell subset identification in Naive B cell-RA-FLS co-culture.** Total cells were initially identified on the FSC-A versus SSC-A plot (1), followed by FSC-H versus FSC-A doublet exclusion to select singlets (2). Live cells were then gated using a viability dye to exclude dead cells (Live/Dead discrimination) (3). Within the live population, B cells were identified and separated based on CD19 versus CD27 expression (4). CD19⁺CD27⁻ (naive B cells) were further analyzed using IgM versus IgD, confirming the naive phenotype as IgM⁺IgD⁺(5). These naive B cells were subsequently assessed for CD90 and Podoplanin (Pdpn) expression (6). CD19⁺CD27⁺ were analyzed using CD38 versus IgG. CD38⁺IgG⁺ double-positive cells were identified as class-switched memory B cells and subsequently assessed for CD90 and Podoplanin expression (7,8). CD19⁻CD27⁻ population was analyzed using IgG versus CD38 (9). Within the CD38⁺IgG⁺ double-positive gate, cells were further assessed for CD138 expression (10). CD138⁺ cells were designated as short-lived plasma-like cells and subsequently analyzed for CD90 and Podoplanin expression (11). Additionally, within the CD19⁻CD27⁻ compartment, the CD38⁻IgG⁻ population was assessed for CD90 and Podoplanin expression to identify additional subsets (12). At late time points in co-culture, the populations gated as naive B cells, class-switched memory B cells, and short-lived plasma-like cells, thus represent B cell-derived CD90⁺podoplanin⁺ aggregates defined by their retained B cell markers, rather than strictly isolated lineage-pure subsets. Consistent gating strategies were applied across all samples. Data were acquired on a CytoFLEX LX cytometer (Beckman Coulter) and analyzed in FlowJo v10.8.1.


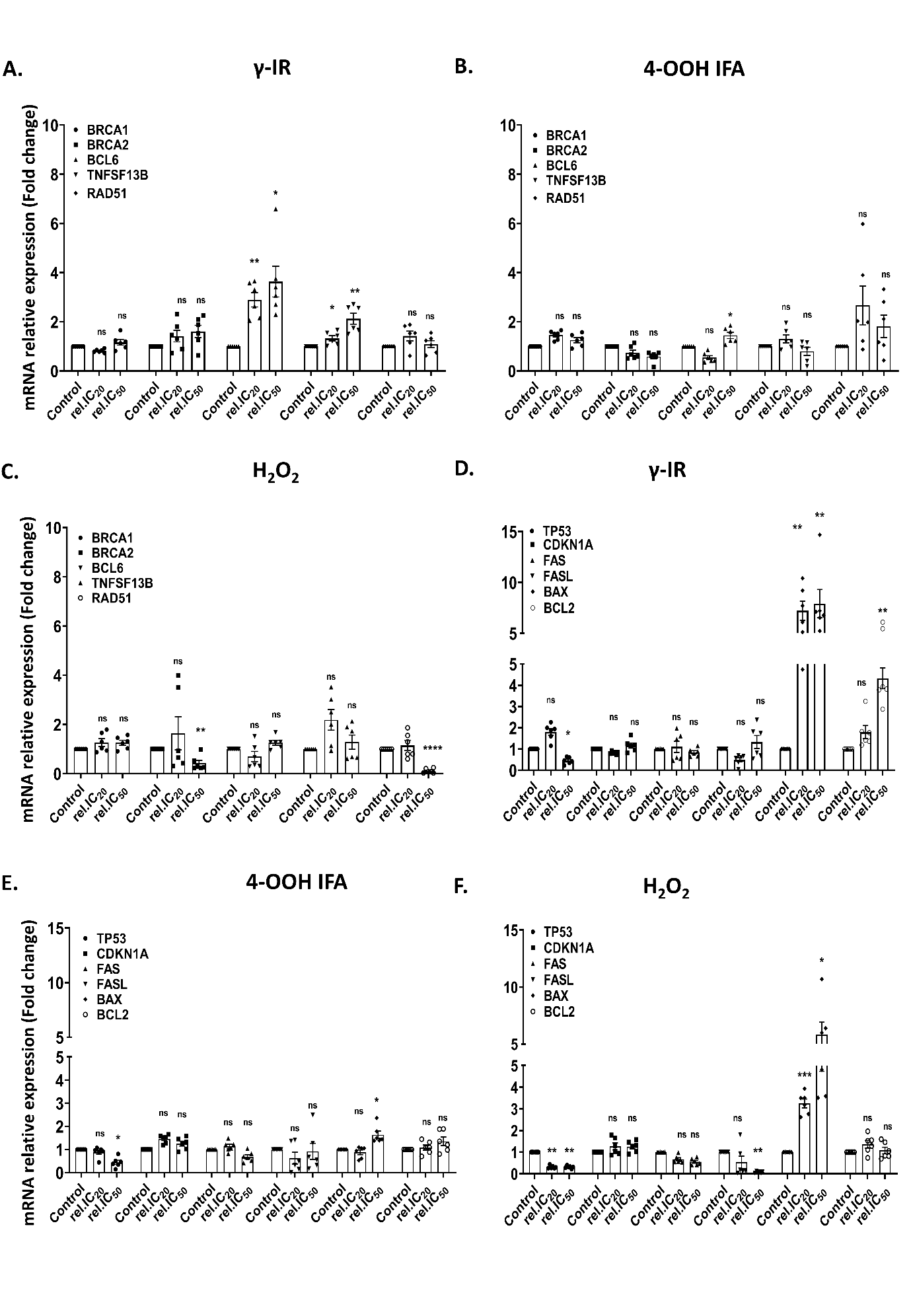


**Supplementary Figure 6. DNA damage response gene expression in naive B cell-RA‑FLS co-cultures at 24 h following genotoxic stress.** Bar charts show fold change (ΔΔCt) mRNA relative to time-matched untreated control in whole naive B cell-RA‑FLS co‑cultures after a single rel.IC₂₀ or rel.IC₅₀ dose/concentration of γ‑IR (A, D), 4‑OOH IFA (B, E), or H₂O₂ (C, F), measured at 24 h post treatment. In all panels, rel. IC₂₀ and rel.IC₅₀ denote relative effect levels that are used as sublethal benchmarks, as defined in the Methods. Panels (A-C) show relative mRNA expression of DNA damage repair and B cell regulatory genes (BRCA1, BRCA2, BCL6,  TNFSF13B, RAD51) in whole naive B cell-RA‑FLS co-cultures following γ‑IR (A), 4‑OOH IFA (B), or H₂O₂ (C) exposure. Panels (D-F) show relative mRNA expression of cell‑cycle checkpoint and apoptosis-related genes (TP53, CDKN1A, FAS, FASL, BAX, BCL2) following γ‑IR (D), 4‑OOH IFA (E), or H₂O₂ (F) exposure. Rel.IC₂₀/IC₅₀ doses/concentrations correspond to viability thresholds defined in Figure 1 (Supplementary Table 2). All data are presented as mean ± SEM with one value per donor and dose/concentration, expressed as percentage or fold change relative to the corresponding untreated control. Group differences versus untreated control were analyzed using repeated‑measures one-way ANOVA with Dunnett’s post‑hoc test; *p ≤ 0.05, **p ≤ 0.01, ***p ≤ 0.001, ****p ≤ 0.0001, ns, not significant. Naive B cell-RA‑FLS co‑cultures: N = 6 donors, n = 1


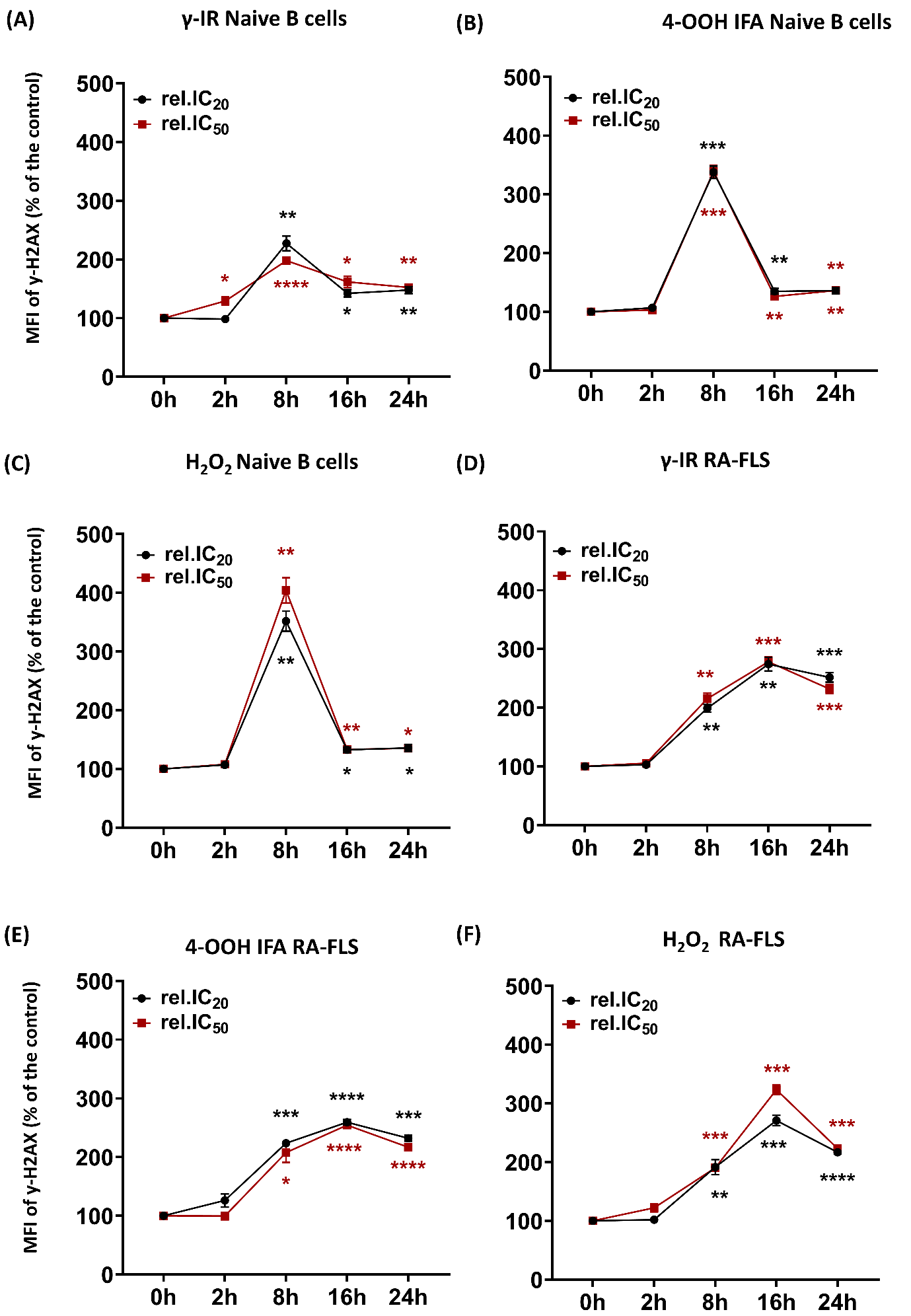


**Supplementary Figure 7. γ‑H2AX dynamics in naive B cell-RA‑FLS co-cultures at rel.IC₂₀ and rel.IC₅₀.** Naive B cell–RA‑FLS co‑cultures were exposed once to rel.IC₂₀ or rel.IC₅₀ concentrations of γ‑irradiation (γ‑IR), 4‑hydroperoxyifosfamide (4‑OOH IFA), or hydrogen peroxide (H₂O₂) as defined in Figure 1 (Supplementary Table 2), and γ‑H2AX MFI was quantified by flow cytometry at 0, 2, 8, 16, and 24 h. In all panels, rel. IC₂₀ and rel.IC₅₀ denote relative effect levels that are used as sublethal benchmarks, as defined in the Methods. Panels (A-C) depict time courses of γ‑H2AX median fluorescence intensity (MFI; % of time‑matched untreated control) in naive B cells at rel.IC₂₀ following γ‑IR (A), 4‑OOH IFA (B), or H₂O₂ (C); panels (D-F) show the corresponding MFI in RA‑FLS at relative IC₅₀ for the same treatments. Naive B cells and RA‑FLS were identified within the co‑cultures by forward/side scatter and surface marker based gating, as detailed in Supplementary Figure 3. All data are presented as mean ± SEM with one value per donor and dose/concentration, expressed as percentage or fold change relative to the corresponding untreated control. Group differences versus untreated control were analyzed using repeated‑measures one-way ANOVA with Dunnett’s post‑hoc test; *p ≤ 0.05, **p ≤ 0.01, ***p ≤ 0.001, ****p ≤ 0.0001, ns, not significant. Naive B cell-RA‑FLS co‑cultures: N = 4 donors, n = 1

**
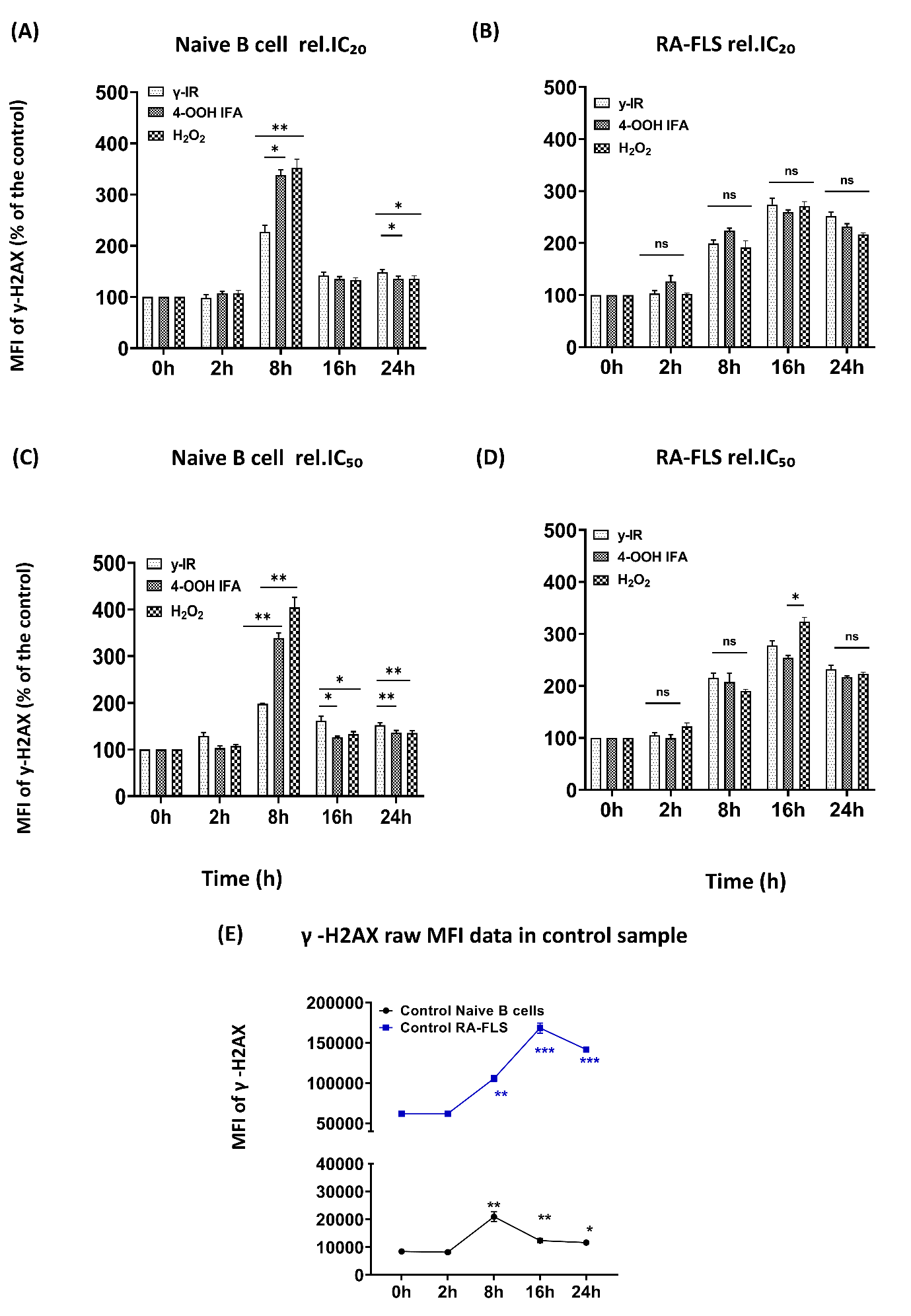
**

**Supplementary Figure 8: Treatment-specific γ‑H2AX responses in naive B cells and RA-FLS over 24 h.** Bar plots depict the median fluorescence intensity (MFI) of phosphorylated histone H2AX (Ser139) in naive (CD19⁺CD27⁻) B cells and RA‑FLS within co-cultures from 0 to 24 h following genotoxic exposure. Panels (A-B) show time point γ‑H2AX MFI in naive B cells (A) and RA‑FLS (B) after treatment with γ‑IR, 4‑OOH IFA, or H₂O₂ at relative IC₂₀ concentrations. Panels (C-D) show the corresponding responses in naive B cells (C) and RA‑FLS (D) at relative IC₅₀ concentrations. MFI values are expressed as percentage of the time‑matched untreated control. Panel (E) shows the corresponding raw γ‑H2AX MFI in untreated control co-cultures for naive B cells and RA‑FLS, illustrating baseline signal levels across the 24 h time course. Relative IC₂₀ and IC₅₀ doses/concentrations are as defined in Figure 1 (Supplementary Table 2) .In all panels, rel. IC₂₀ and rel.IC₅₀ denote relative effect levels that are used as sublethal benchmarks, as defined in the Methods. Cell populations were identified by forward/side scatter and surface marker gating as outlined in Supplementary Figure 3. All data are presented as mean ± SEM with one value per donor and dose/concentration, expressed as percentage or fold change relative to the corresponding untreated control. Group differences for treatment conditions (Panels A-D) were analyzed using a repeated‑measures one-way ANOVA with Bonferroni’s post-hoc test, while baseline changes over time (Panel E) were evaluated using a repeated-measures one-way ANOVA with Dunnett’s post‑hoc test against the 0 h control; *p ≤ 0.05, **p ≤ 0.01, ***p ≤ 0.001, ****p ≤ 0.000, ns, not significant. Naive B cell-RA‑FLS co‑cultures: N = 4 donors, n = 1

**
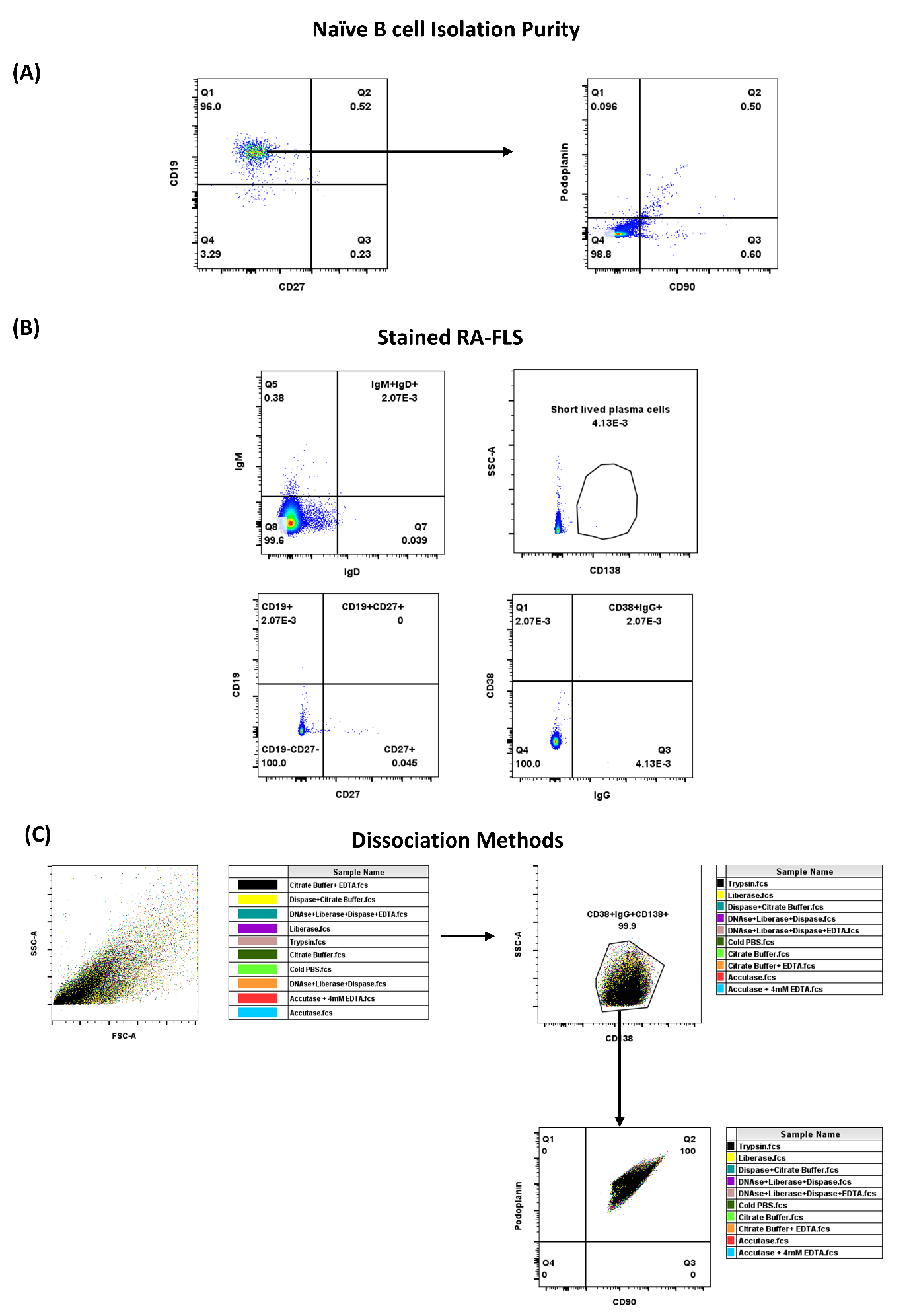
**

**Supplementary Figure 9. Naive B cell isolation purity, RA‑FLS phenotype confirmation, and co-culture dissociation strategy.** Panel (A) shows representative flow cytometry dot plots confirming naive B cell isolation purity following negative selection. Left plot: CD19⁺CD27⁻ gating identifies naive B cells (96.0% purity). Right plot: Podoplanin vs. CD90 exclusion confirms absence of RA-FLS and other contaminating populations. Panel (B) shows phenotypic characterization of cultured RA‑FLS used in co-culture experiments. Top plots: RA‑FLS are negative for B cell markers (IgM⁻IgD⁻, <0.4%) and short‑lived plasma like cells markers (SSC‑A vs. CD138). Bottom plots: RA‑FLS lack CD19, CD27 expression (CD19⁻CD27⁻: 100%) and CD38, IgG (CD38⁻IgG⁻: 100%), confirming absence of B lineage contamination. Panel (C) illustrates the dissociation and gating strategy used to separate naive B cells from RA‑FLS in co-cultures prior to flow cytometry analysis. Left: FSC‑A vs. SSC‑A scatter distinguishes cell populations; middle: CD38⁺IgG^+^CD138⁺ gate (99.9%) enriches for B lineage cells. Multiple dissociation methods were tested to optimize B cell recovery and viability from adherent RA‑FLS layers, as indicated in the sample legend.

**
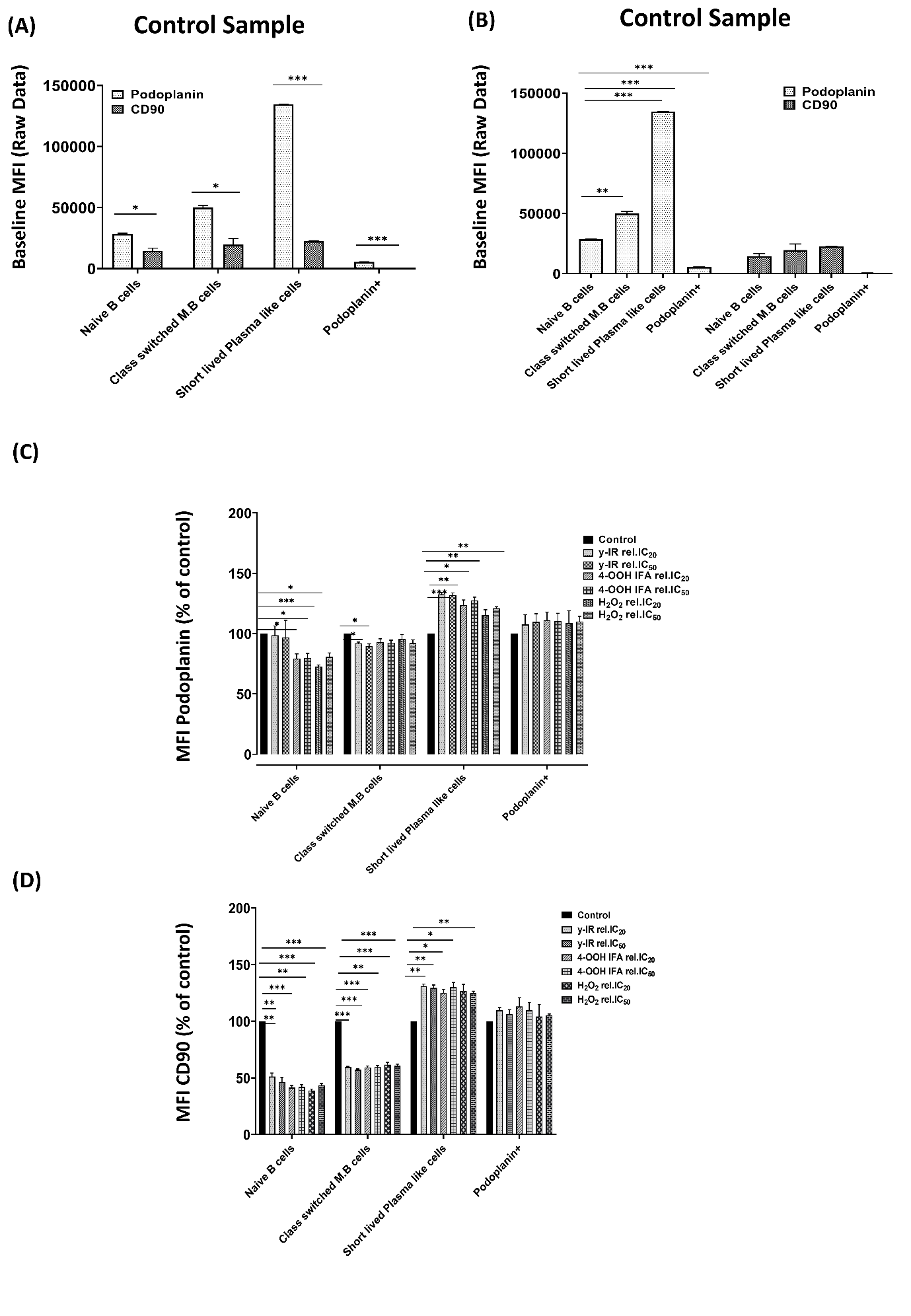
**

**Supplementary Figure 10. Podoplanin and CD90 expression in B cell subsets and effect of genotoxic treatment.** Panels (A-B) show baseline expression of the fibroblast markers podoplanin and CD90 across B cell subsets and RA-FLS populations in control (untreated, stimulated) samples. Bar graphs depict median fluorescence intensity (MFI) of podoplanin and CD90 in naive B cells, class-switched memory B cells, short-lived plasma like cells, and podoplanin⁺ cells.  At this late time point, the populations referred to as naive B cells, class-switched memory B cells, and short-lived plasma like cells represent B cell-derived CD90⁺podoplanin⁺ aggregates defined by their retained B cell markers rather than strictly isolated lineage‑pure subsets. Panels (C-D) show the effect of genotoxic treatment on the expression of podoplanin (C) and CD90 (D) in the same B cell subsets at 9d post-treatment. Bar graphs depict MFI as a percentage of time‑matched to untreated control for each subset following exposure to γ‑IR, 4‑OOH IFA, or H₂O₂ at relative IC₂₀ and IC₅₀ concentrations as defined in Figure 1 (Supplementary Table 2).In all panels, rel. IC₂₀ and rel.IC₅₀ denote relative effect levels that are used as sublethal benchmarks, as defined in the Methods. Naive B cells, class-switched memory B cells, short-lived plasma like cells, and podoplanin⁺ cells were identified by surface marker gating as outlined in Supplementary Figure 5. All data are presented as mean ± SEM with one value per donor and dose/concentration, expressed as percentage or fold change relative to the corresponding untreated control. Baseline group differences (Panels A-B) were analyzed using a repeated-measures one-way ANOVA with Bonferroni’s post-hoc test. The effects of genotoxic treatments relative to the untreated control (Panels C-D) were analyzed using a repeated‑measures one-way ANOVA with Dunnett’s post‑hoc test; *p ≤ 0.05, **p ≤ 0.01, ***p ≤ 0.001, ****p ≤ 0.000, ns, not significant. Naive B cell-RA‑FLS co‑cultures: N = 6 donors, n = 1


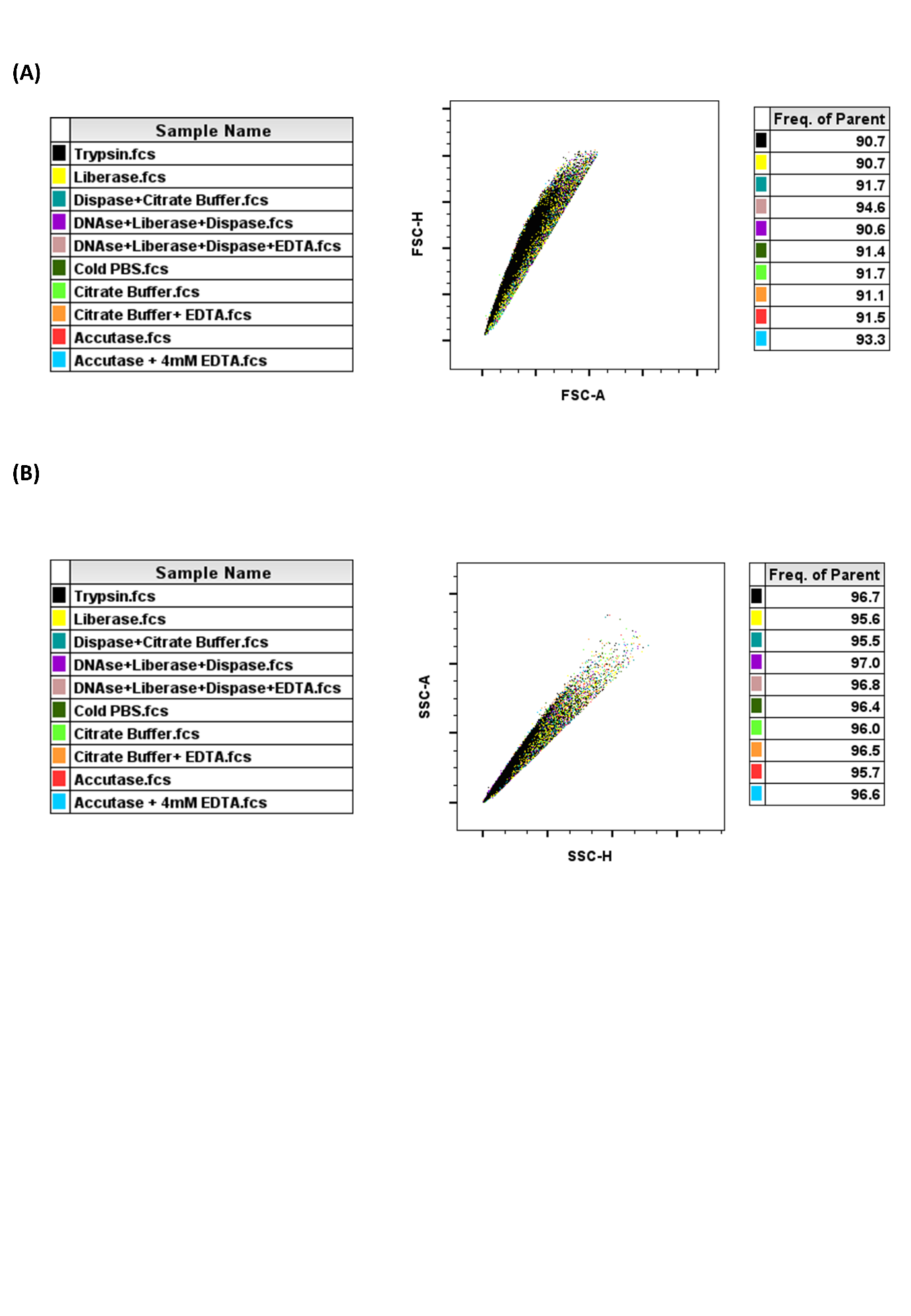


**Supplementary Figure 11. Doublet discrimination and singlet gating in late co‑culture dissociation experiments.** RA‑FLS/naive B cell co‑cultures at day 9 were subjected to different dissociation conditions (trypsin, Liberase, dispase + citrate buffer, DNase + Liberase + dispase, DNase + Liberase + dispase + EDTA, cold PBS, citrate buffer, citrate buffer + EDTA, Accutase, Accutase + 4 mM EDTA). (A) Forward‑scatter area (FSC‑A) versus height (FSC‑H) plots showing the singlet gate applied to all conditions. The legend indicates the proportion of events within the singlet gate (“Freq. of Parent”) for each dissociation condition, which ranged from approximately 90% to 95%. (B) Side‑scatter area (SSC‑A) versus height (SSC‑H) plots with the corresponding singlet gate and frequencies, with approximately 95%–97% of events falling within the singlet gate. These data illustrate that the majority of analysed events meet conventional single‑event criteria by pulse‑shape analysis, although, as discussed in the main text, tightly adherent B cell-RA‑FLS conjugates cannot be fully excluded in strongly aggregated co‑cultures and late readouts are interpreted as measurements on B cell-derived aggregates within this adherent microenvironment.

## Supplementary Tables

**Supplementary Table 1: All used compounds and Antibodies for the study.**

| Antibody/Compound/Kit | Fluorochrome | Species | Company | Catalog Nr. | Final Conc. |
| --- | --- | --- | --- | --- | --- |
| CD19 | efluor 405 | Human | Invitrogen | 48-0199-42 | 1:20 |
| CD27 | VioBright R720 | Human | Miltenyi Biotec | 130-128-404 | 1:50 |
| CD27 | APC | Human | Miltenyi Biotec | 130-113-626 | 1:50 |
| IgD | PE-CF594 | Human | BD Biosciences | 562540 | 1:20 |
| IgM | Alexa Fluor 700 | Human | Biolegend | 314538 | 1:20 |
| IgG | APC-Vio770 |  | Miltenyi Biotec | 130-119-876 | 1:50 |
| CD5 | PE | Human | BD Biosciences | 555353 | 1:20 |
| CD1d | BV786 | Human | BD Biosciences | 743608 | 1:20 |
| CD11c | BV605 | Human | BD Biosciences | 563929 | 1:20 |
| CD38 | PE-Cy5 | Human | BD Biosciences | 303508 | 1:20 |
| CD138 | PE-Vio770 | Human | Miltenyi Biotec | 130-135-362 | 1:50 |
| CD90 | PerCP-Vio700 | Human | Miltenyi Biotec | 130-114-864 | 1:50 |
| Podoplanin | PerCP-Cy5.5 | Human | Biolegend | 337012 | 1:20 |
| Zombie UV |  | Human | Biolegend | 423107 | 1:1000 |
| y-H2AX | Alexa Fluor 488 | Human | BD Biosciences | 560445 | 1:20 |
| KI-67 | FITC | Human | Miltenyi Biotec | 130-117-691 | 1:50 |
| PI | - | Human | Miltenyi Biotec | 130-093-233 | 1:100 |
| Annexin V/PI kit | FITC | Human | Miltenyi Biotec | 130-092-052 |  |
| Fcr-blocking reagent |  | Human | Miltenyi Biotec | 130-059-901 | 1:5 |
| BD Horizon™ Brilliant Stain Buffer |  |  | BD Biosciences | 566349 |  |
| y-IR : Gammacell 100 Elite radiation machine |  |  | Nordion International |  |  |
| 4-OOH IFA |  |  | Niomech IIT GmbH | D-18851 |  |
| H₂O₂ |  |  | Sigma-Aldrich | [7722-84-1](https://www.sigmaaldrich.com/DE/en/search/7722-84-1?focus=products&page=1&perpage=30&sort=relevance&term=7722-84-1&type=cas_number) |  |
| AffiniPure® Goat Anti-Human IgM, Fc_5μ_ fragment specific |  |  | Jackson ImmunoResearch | 109-005-043 | 10 µg/mL |
| CpG ODN 2006 |  | Human | Invivogen | tlrl-2006-1 | 5 µg/mL |
| Human IL-10 ELISA set |  | Human | BD Biosciences | 555157 |  |
| Human IFN-y ELISA set |  | Human | BD Biosciences | 555142 |  |
| Human APRIL/TNFSF13 DuoSet ELISA |  | Human | R&D | DY884B |  |
| Human TNF-alpha DuoSet ELISA |  | Human | R&D | DY210-05 |  |
| Human TACI/TNFRSF13B DuoSet ELISA |  | Human | R&D | DY174 |  |
| Human IL-4 DuoSet ELISA |  | Human | R&D | DY204 |  |
| Human IgM ELISA Antibody Pair Kit |  | Human | StemCell | #01995A |  |
| Human IgG ELISA Antibody Pair Kit |  | Human | StemCell | #01994A |  |
| Human IgA ELISA Antibody Pair Kit |  | Human | StemCell | #01992A |  |
| Human IgE ELISA Antibody Pair Kit |  | Human | StemCell | #01993A |  |
| Dispase II, powder |  |  | ThermoFisher Scientific | 17105041 |  |
| DNase I Solution (1 mg/mL) |  |  | Stemcell | 07900 |  |
| Liberase^™^ TM Research Grade |  |  | Sigma-Aldrich | 5401127001 |  |
| 0.5 M EDTA |  |  | Invitrogen | 15575020 |  |
| Trypsin-EDTA (0,05 %), phenol red |  |  | ThermoFisher Scientific | 25300062 |  |
| Accutase^®^ solution |  |  | Sigma-Aldrich | A6964-100ML |  |
| CellTrace™ CFSE Cell Proliferation Kit |  |  | ThermoFisher Scientific | C34554 |  |

**Supplementary Table 2: Relative IC₅₀ values (24 h and 8 d) unless stated otherwise such as y-Irradiation rel.IC_20_ , 4-hydroperoxy ifosfamide- rel.IC_20_ , or Hydrogen Peroxide- rel.IC_20_ which refers to relative IC_20_ values and not relative IC_50_ derived from the four-parameter fits in main Figure 1.**

| Time | Treatment | Cell Type | Relative IC_50_ (μM/Gy) |
| --- | --- | --- | --- |
| **24h** | y-Irradiation | Memory B cells | 1.4 Gy |
| **24h** | y-Irradiation | Naïve B cells | 1.3 Gy |
| **24h** | 4-hydroperoxy ifosfamide | Memory B cells | 5 μM |
| **24h** | 4-hydroperoxy ifosfamide | Naïve B cells | 7.8 μM |
| **24h** | Hydrogen Peroxide | Memory B cells | 35 μM |
| **24h** | Hydrogen Peroxide | Naïve B cells | 78 μM |
| **24h** | y-Irradiation- rel.IC_20_ | Naïve B cells | 0.58 Gy |
| **24h** | 4-hydroperoxy ifosfamide- rel.IC_20_ | Naïve B cells | 3.5 μM |
| **24h** | Hydrogen Peroxide- rel.IC_20_ | Naïve B cells | 24 μM |

**Supplementary Table 3: List of utilized primers. All primer sequences are listed from 5′ to 3′ and are identical to those reported previously in Bruci et al., 2026 (1)**

| Gene | Forward Primer (5'-3') | Reverse Primer (5'-3') |
| --- | --- | --- |
| *ATM* | *TTACGGGTGTTGAAGGTGTCT* | *GGATTCATGGTCCAGTCAAAG* |
| *APEX1* | *CTGCCTGGACTCTCTCATCAATAC* | *CCTCATCGCCTATGCCGTAAG* |
| *XRCC6* | *AAAAGACTGGGCTCCTTGGT* | *TGTGGGTCTTCAGCTCCTCT* |
| *XRCC5* | *CGACAGGTGTTTGCTGAGAA* | *GAATCACATCCATGCTCACG* |
| *RAD50* | *CTTGGATATGCGAGGACGAT* | *CGCATTGAAGGTCGAAGACC* |
| *RAD51* | *GCCACCGCCCTTTACAGAACA* | *TGGGATCAGCAGCAAACATCG* |
| *BRCA1* | *CCACAGATCAACCTGGAATGG* | *GTAGAGTGCTACACTGCTCA* |
| *BRCA2* | *TTCTGAGGTGGACCTAATAGG* | *TGATTTGGATTCTGGTCGCC* |
| *FAS* | *GACCCTCCTACCTCTGGTTCTT* | *TGTGCAGTCCCTAGCTTTCC* |
| *FASLG* | *TCTACCAGCCAGATGCACAC* | *CTTGAGTTGGACTTGCCTGT* |
| *BAX* | *GCCCTTTTGCTTCAGGGTTT* | *TCCAATGTCCAGCCTTTG* |
| *BCL2* | *GAAGCATACCCGTTTAGC* | *CGAGAACTGGGAGAAGAA* |
| *TP53* | *TTCCGAGAGCTGAATGAGGC* | *AATGTCAGTCTGAGTCAGGCC* |
| *CDKN1A* | *TACATCTTCTGCCTTAGT* | *TCTTAGGAACCTCTCATT* |
| *PDCD1* | *CGTGACTTCCACATGAGCGT* | *CTGGCTCCTATTGTCCCTCGT* |
| *BCL6* | *AGGCCGGACACCAGGTTTTG* | *GCTCTAAACTGCTCACGGCT* |
| *TNFSF13B* | *GTGAAACACCAACTATACAAAAAGG* | *GTTTTGCAATGCCAGCTGAA* |
| *BACH2* | *CGTTCGTTCACATAGCTCCCA* | *TCAGTGAGTGTCCACCTTGTTC* |
| *XBP1* | *TCTGGAGCTATGGTGGTGGT* | *GTTTCCTCCTCAGCGCCTT* |
| *IRF4* | *CCCAGCTTGTGAAAATGGTTG* | *ACCTTATGCTTGGCTCTGTGG* |
| *PAX5* | *ATTTCACGGTGCCTTCGGAC* | *TCACTCCTCCATGTCCTGTC* |
| *PRDM1* | *CTCCAGTGTTGCGGAGAGG* | *GGGGCAGCCAAGGTCG* |
| *AICDA* | *CGCATCCTTTTGCCCCTGTA* | *GCCAGACCTGTGTTCCTTCT* |
| *IGHM* | *GCTGAGGCAAAGGAGTCTG* | *TGGTCTGCTTCAGTGGCG* |
| *IGHA1* | *ACCATGCAGGAGAAGGTGTC* | *TCACTTGCACTGCTGCCTAC* |
| *IGHG1* | *GCAGCCGGAGAACAACTACA* | *TGGTTGTGCAGAGCCTCGAT* |
| *IL10* | *GGAAGCCAGGATCACCAACA* | *CCTTCCATGCTTTGGGGTTG* |
| *TLR9* | *GCCAGACCCTCTGGAGAA* | *GGCACAGTCATGATGTTGTTGTA* |
| *r18S* | *TAACCCGTTGAACCCCATT* | *CCATCCAATCGGTAGTAGCG* |

**References:**

1. Bruci D, Lowin T, Fritz G, Pongratz G. Sublethal DNA damage switches off B cell effector programs in an RA-FLS-PBMC co-culture. Cell Death Discov. 2026;12(1).
